# A hierarchy of manganese competition and entry in organotypic hippocampal slice cultures

**DOI:** 10.1002/nbm.4476

**Published:** 2021-02-03

**Authors:** Emily Petrus, Galit Saar, Alexia Daoust, Steve Dodd, Alan P. Koretsky

**Affiliations:** ^1^ Laboratory of Functional and Molecular Imaging National Institute of Neurological Disorders and Stroke, National Institutes of Health Bethesda Maryland USA

**Keywords:** contrast enhancement, hippocampus, manganese, MEMRI, MRI

## Abstract

Contrast agents improve clinical and basic research MRI. The manganese ion (Mn^2+^) is an essential, endogenous metal found in cells and it enhances MRI contrast because of its paramagnetic properties. Manganese‐enhanced MRI (MEMRI) has been widely used to image healthy and diseased states of the body and the brain in a variety of animal models. There has also been some work in translating the useful properties of MEMRI to humans. Mn^2+^ accumulates in brain regions with high neural activity and enters cells via voltage‐dependent channels that flux calcium (Ca^2+^). In addition, metal transporters for zinc (Zn^2+^) and iron (Fe^2+^) can also transport Mn^2+^. There is also transfer through channels specific for Mn^2+^. Although Mn^2+^ accumulates in many tissues including brain, the mechanisms and preferences of its mode of entry into cells are not well characterized. The current study used MRI on living organotypic hippocampal slice cultures to detect which transport mechanisms are preferentially used by Mn^2+^ to enter cells. The use of slice culture overcomes the presence of the blood brain barrier, which limits inferences made with studies of the intact brain in vivo. A range of Mn^2+^ concentrations were used and their effects on neural activity were assessed to avoid using interfering doses of Mn^2+^. Zn^2+^ and Fe^2+^ were the most efficient competitors for Mn^2+^ uptake into the cultured slices, while the presence of Ca^2+^ or Ca^2+^ channel antagonists had a more moderate effect. Reducing slice activity via excitatory receptor antagonists was also effective at lowering Mn^2+^ uptake. In conclusion, a hierarchy of those agents which influence Mn^2+^ uptake was established to enhance understanding of how Mn^2+^ enters cells in a cultured slice preparation.

## Graphical abstract

The manganese ion (Mn2+) is an endogenous, paramagnetic cation that is used as a contrast agent for in vivo MRI. Manganese‐enhanced MRI (MEMRI) is used in preclinical models because of its ability to highlight cytoarchitecture, enter active cells and trace neural connections. Manganese enters cells through metal and/or cation transporters, and activity‐dependent calcium channels. Hippocampal slice cultures were used to measure the relative contribution of each uptake pathway and to describe a hierarchy of entry routes into hippocampal subregions.

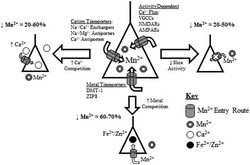

**Abbreviations used**
aCSF: artificial cerebrospinal fluid
AD: Alzheimer's disease
AMPA receptor: α‐amino‐3‐hydroxy‐5‐methyl‐4‐isoxazolepropionic acid (ionotropic glutamate receptor)
APV: (2R)‐amino‐5‐phosphonovaleric acid, or (2R)‐amino‐5‐phosphonopentanoate)
MEMRI: manganese‐enhanced magnetic resonance imaging
MK‐801: NMDA receptor antagonist
NBQX: AMPA receptor antagonist
NMDA receptor: N‐Methyl‐d‐aspartic acid (ionotropic glutamate receptor)
OHSC: organotypic hippocampal slice culture
TTX: tetrodotoxin (Na^+^ channel blocker)
VGCC: voltage‐gated calcium channel
verapamil: VGCC blocker

## 1 INTRODUCTION

Manganese‐enhanced MRI (MEMRI) has been widely used to image healthy and diseased states of the body and the brain in a variety of animal models.^1^, ^2^, ^3^, ^4^, ^5^, ^6^ There has also been some work in translating the useful properties of MEMRI to humans.^4^, ^7^, ^8^ Mn^2+^ accumulates in brain regions with high neural activity and enters cells via voltage‐dependent channels that flux calcium (Ca^2+^).^9^, ^10^, ^11^, ^12^, ^13^ In addition, metal transporters for zinc (Zn^2+^) and iron (Fe^2+^) can also transport Mn^2+^.^14^, ^15^ There is also transfer through channels specific for Mn^2+^.^16^, ^17^ Manganese ions are essential for life and proper brain function.^18^, ^19^, ^20^ Mn^2+^ is useful as a contrast agent in magnetic resonance imaging (MRI) due to its paramagnetic properties.^21^, ^22^, ^23^ Mn^2+^ accumulates in tissue and shortens the spin–lattice relaxation time (T_1_) and spin–spin relaxation time (T_2_) of water molecules. Contrast enhancement in T_1_‐weighted (T_1W_) images is typically used to follow changes in Mn^2+^ levels^21^, ^24^ because higher doses are required to shorten typical tissue T_2_s, which are much shorter than T_1_._4_ Systemic administration of Mn^2+^ yields accumulation in the heart, liver, kidneys and pancreas.^25^, ^26^, ^27^ On a slower timescale, Mn^2+^ also accumulates in specific brain tissues, including cortex, olfactory bulb, hippocampus, cerebellum and the pituitary gland.^6^, ^28^, ^29^, ^30^, ^31^ This pattern of accumulation has been useful for defining cytoarchitecture in a number of brain regions with MRI.^2^, ^4^, ^25^ Mn^2+^ also defines neural pathways as an anterograde tracer when administered to peripheral sensory systems or by stereotaxic cranial injection.^1^, ^3^, ^32^ Finally, Mn^2+^ can accumulate in excitable cells such as neurons, cardiac cells, rapidly dividing carcinogenic cells and pancreatic β‐cells based on the membrane potential dependent transport through L‐type voltage‐gated calcium channels (VGCCs).^2^, ^5^, ^33^ These three useful properties of MEMRI have led to its widespread use in animal models of normal and diseased tissue like those found in Alzheimer's disease and schizophrenia.^3^, ^34^, ^35^

Despite its widespread use there is no quantitative understanding of the biological factors that control Mn^2+^ uptake and distribution within cells. This is especially true of the brain where Mn^2+^ entry through the blood brain barrier (BBB) must be considered. The hippocampus preferentially accumulates Mn^2+^ and offers an excellent opportunity for studying the mechanisms that underlie MEMRI.^36^, ^37^, ^38^ Mn^2+^ accumulates in the hippocampus of young and adult animals^39^ with specific accumulation in the dentate gyrus and CA3 hippocampal subregions.^28^, ^29^, ^40^ Indeed, the ability of MEMRI to give cytoarchitectural information is due to the biological pattern of region‐specific accumulation of Mn^2+^. In addition to subregions of the hippocampus, layers of the cerebellum, olfactory bulb and cortex experience varied Mn^2+^ accumulation in vivo.^41^ There is currently no model for why these subregions and layers accumulate different amounts of Mn^2+^. It is likely that the density, activity and intrinsic transport and retention properties of various regions contribute to the MEMRI contrast in the brain.^42^

The mechanisms of how Mn^2+^ enters cells, where and how much is retained, and how it is transported along synaptic pathways and crosses synapses is not understood. There are Mn^2+^‐specific transporters that enable cells to carry out functions requiring this essential metal. Mn^2+^ can also enter cells like Ca^2+^ through VGCCs and glutamatergic NMDA and/or α‐amino‐3‐hydroxy‐5‐methyl‐4‐isoxazolepropionic acid (ionotropic glutamate receptor) (AMPA) receptors.^9^, ^10^, ^11^, ^43^, ^44^ Other cation channels such as sodium (Na^+^)/Ca^2+^ exchangers, Na^+^/Mg^2+^ antiporters and activated Ca^2+^ antiporters also allow Mn^2+^ entry and efflux from cells. These entry mechanisms likely explain the activity‐dependent accumulation in a variety of tissues including brain observed with MEMRI. In addition, Mn^2+^ can be transported via systems that carry other metals such as iron (Fe^2+^) and zinc (Zn^2+^). For example, transferrin‐bound Mn^2+^ can bind to transferrin receptors and be taken up in a manner similar to iron.^45^ The divalent metal transporters (DMTs) 1‐6, zinc transporters ZIP8, and others,^10^, ^12^, ^13^, ^14^, ^46^, ^47^, ^48^, ^49^ are also known to transport Mn^2+^ with various affinities. DMTs are known to have differential sensitivity to transporting metal ions, including Fe^2+^, Zn^2+^, copper (Cu^2+^) and cobalt (Co^2+^), as well as Mn^2+^. Thus, Mn^2+^ can mimic divalent cations such as Ca^2+^ and bind to sites for Mn^2+^, Mg^2+^, Zn^2+^ and Fe^2+^. It is likely the properties used in MEMRI are a complex mixture of processes depending on Mn^2+^ concentration and the tissue in which it accumulates.

After cellular entry in neurons, Mn^2+^ is anterogradely trafficked in a microtubule‐dependent manner until it arrives at a presynaptic terminal.^43^ Mn^2+^ is then thought to be packaged into and released with vesicles and subsequentially taken up by postsynaptic neurons, thus enabling real‐time monitoring of neuronal transport and connectivity.^2^, ^39^ In summary, the biological properties of Mn^2+^ are driven by a wide variety of mechanisms that enable its transport throughout the nervous system. Although there are a variety of entry routes available to Mn^2+^, quantitative understanding of how Mn^2+^ enters tissue and progresses along afferent routes is lacking.

The purpose of this study was to evaluate how Mn^2+^ accumulates inside hippocampal cells. This was performed in organotypic hippocampal slice cultures (OHSCs) from neonatal rats, which circumvented the presence of the BBB and enabled visualization of direct cell entry. Optimal Mn^2+^ concentration and duration of exposure were determined to enhance contrast but not significantly impact slice viability and activity. The slices were then exposed to substrates designed to alter the ability of Mn^2+^ to enter cells by transporters or cation channels. These results demonstrated that Mn^2+^ accumulation at the concentrations and timescales used for loading depended largely on metal transporters and, to a lesser extent, calcium and other activity channels.

## 2 EXPERIMENTAL METHODS

### 2.1 Organotypic hippocampal slice culture

All animal experiments were performed in accordance with NIH guidelines and were approved by the Animal Care and Use Committee of the National Institute of Neurological Disorders and Stroke, National Institutes of Health (Bethesda, MD, USA). OHSCs were generated from postnatal day 6 Sprague Dawley rat pups, as previously described.^50^ In brief, both hippocampi were isolated, cut into 300‐μm sections with a McIlwain tissue chopper (Ted Pella, CA, USA) and plated onto a cell culture insert (Corning, Falcon, Fisher Scientific, MD, USA) in Hank's Salts Essential Medium supplemented with 25% Hank's Balanced Salt Solution, 25% horse serum, 1 mM glutamine, 30 mM glucose 45%, 1 mM pyruvate, 0.5 mM ascorbate (Corning) and 2.5% N3 solution (solution made in phosphate buffered saline supplemented with 1 mg/ml BSA, 10 mg/ml transferrin, 10 mM putrescine, 3 μM selenium, 1 μg/ml triiodothyronine, 0.5 mg/ml insulin, 0.2 μM progesterone and 0.2 μg/ml corticosterone). Twenty‐four hours after culture, the horse serum concentration was reduced from 25% to 5%. Thereafter, cultures were fed every 2–3 days with low serum concentration medium. Cultures were maintained for 6–7 days prior to MRI.

### 2.2 Manganese, metals and drugs incubation

A summary of all the metals and drugs used in this study and the incubation times are listed in Table S1. Experimental time courses can be found in Figure S1.

Manganese solutions (M8054; Sigma, St. Louis, MO, USA), 0–150 μM, were initially studied and manganese solutions were added to medium culture of OHSCs at distinct time points (1–24 hours) prior to imaging. Based on having excellent MRI enhancement and low cell damage rates, 25 μM Mn^2+^ concentration was used in subsequent experiments. Thereafter, Mn^2+^ was added to the OHSC culture medium 2 hours before imaging but Mn^2+^ and all reagents were washed out just prior to imaging.

Mn^2+^ uptake through Ca^2+^ channels was studied by adding 3 or 6 mM Ca^2+^ to the culture medium simultaneously with Mn^2+^, 2 hours prior to imaging. Forty μM of verapamil (Sigma), a Ca^2+^ channel antagonist, was added to the medium 2 hours before Mn^2+^ (4 hours prior to imaging).

Mn^2+^ cell uptake due to glutamate receptor activity was studied by adding antagonists to the culture medium. The AMPA antagonist NBQX (1 μM; Sigma) and/or the NMDA antagonist MK‐801 (1 μM; Sigma) were added 2 hours before Mn^2+^ (4 hours prior to imaging) (Figure S1C).

Mn^2+^ uptake due to activity was studied by adding tetrodotoxin (TTX; 1 μM; Sigma) alone or in addition to glutamate antagonists (as described above) to the medium culture. TTX was added at the same time as Mn^2+^ (2 hours prior to imaging).

Mn^2+^ uptake through divalent metal transporters was studied by adding Zn^2+^ (1 mM; Sigma), Fe^2+^ (1 mM; Sigma) or both Fe^2+^ and Zn^2+^ to the medium culture simultaneously with Mn^2+^ (2 hours prior to imaging). The ferromagnetic contrast generated by Fe^2+^ in MRI images was accounted for by incubating separate OHSCs with 1 mM Fe^2+^ and correcting MRI measurements.

### 2.3 Manganese‐enhanced MRI

For MEMRI, the slices were removed from the cell culture insert. The membrane insert was cut around the slice and rinsed in artificial cerebrospinal fluid (aCSF; 124 mM NaCl_2_, 5 mM KCl, 1.23 mM NaH_2_PO_4_, 26 mM NaHCO_3_, 10 mM dextrose, 1.5 mM MgCl_2_ and 2.5 mM CaCl_2_ in water), placed in a perfusion chamber then fixed with a plastic anchor (Figure S1). To keep the cultures alive during imaging, slices were continuously perfused with the aCSF at 0.9 μL/min, and bubbled with 95% O_2_ and 5% CO_2_ at 37.5 ± 0.5°C. On those rare occasions when microbubbles were observed, they were not included in the ROI measurements.

Imaging was performed on an 11.7 T/30 cm horizontal bore magnet (Magnex Scientific, Oxford, UK) with an Avance III console (Bruker Biospin, Billerica, MA, USA), using a volume transmit coil and a surface coil (diameter = 1 cm) for detection. 3D imaging was performed to determine the slice position in the MRI. 3D T_1w_ images were then acquired with a fast low angle shot (FLASH) sequence, with 50 μm isotropic resolution, 25^o^ pulse, FOV = 19.2 × 19.2 × 3.2 mm^3^, matrix size = 384 × 384 × 64, TR/TE = 25/4.7 ms, and a total scan time of approximately 11 minutes. One to two MRI slices from the center of the hippocampal slices were selected for ROI analysis. T_1_ mapping is more quantitative, as T_1w_ imaging is not linearly proportional to Mn over all the concentrations used in our experiments. However, T_1w_ images and Mn concentrations were roughly linearly within the range in which most experiments were performed. Further, the goal of the experiments was to order the routes of Mn uptake in slices instead of precisely calculating the Mn levels, thus T_1w_ imaging was used instead of T1 or R1 mapping. Further, T_1w_ imaging reduces scan time to maintain slice viability and can increase resolution to detect Mn^2+^ signal in hippocampal subregions.

### 2.4 MRI data analysis

**FIGURE 1:**
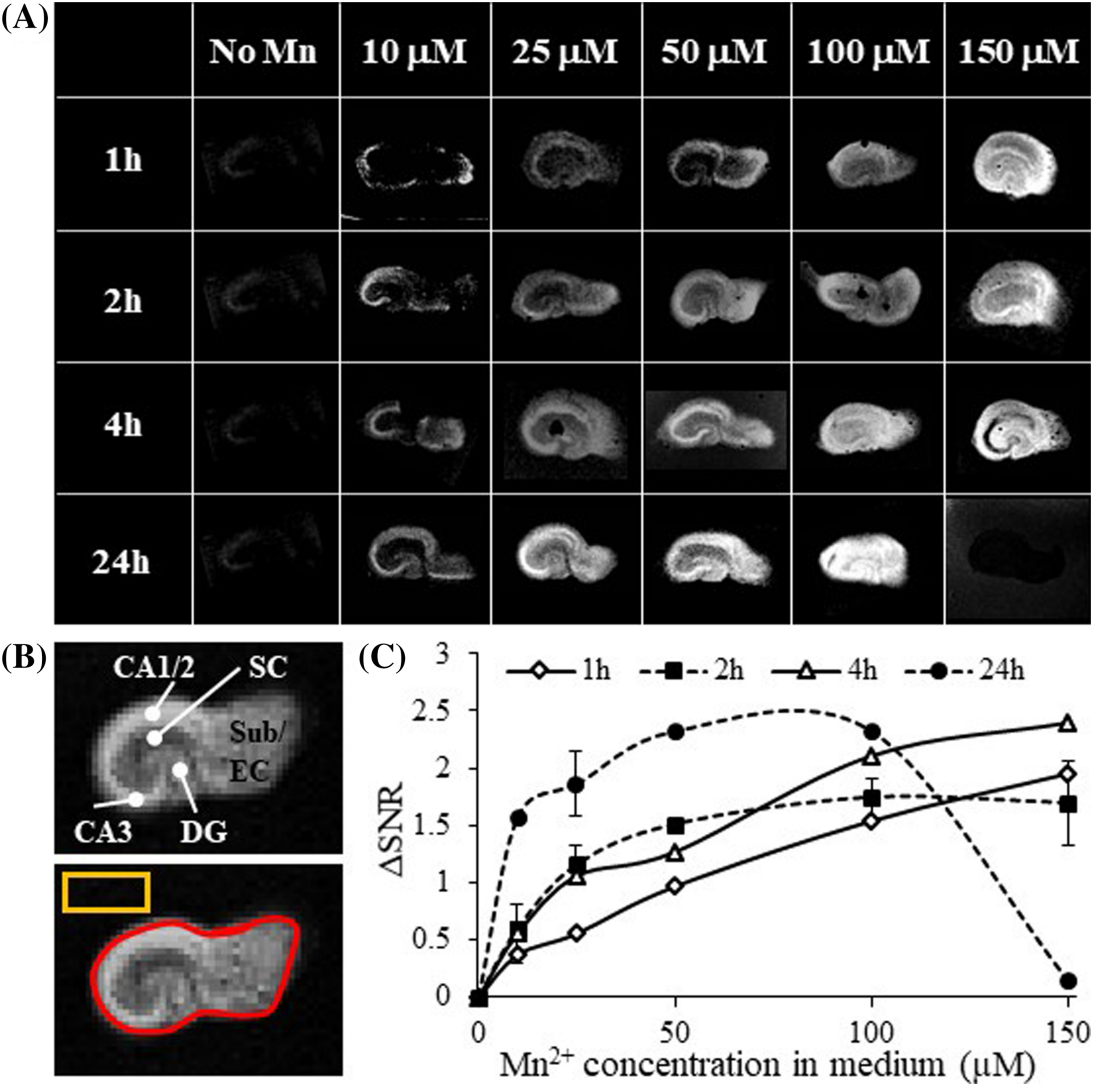
Optimal contrast detected with minimal Mn^2+^ concentrations and incubation times. A, T_1_‐weighted images of OHSCs after Mn^2+^ incubation. Almost no contrast was observed in the absence of Mn^2+^. B, Depiction of OHSC ROIs of whole slice, hippocampal subregions, and background measurement (rectangle). C, ΔSNR curve for all Mn^2+^ concentrations at different incubation times; n = 3–6 slices, values are average ± standard deviation (SD). The ΔSNR increased with increased Mn^2+^ concentration and incubation time. Incubation for 24 hours showed a saturation plateau between 50 and 100 μM Mn^2+^, with a steep drop in ΔSNR at 150 μM Mn^2+^

Data analysis was performed using Medical Image Processing, Analysis, and Visualization software (NIH; http://mipav.cit.nih.gov). ROIs were manually drawn around the slice and in five different areas using a rat brain atlas^51^ as a visual reference: dentate gyrus (DG), Cornu Ammonis (CA3 and CA1/2), Schaffer collaterals (SC) and subiculum/entorhinal cortex (Sub/EC) (Figure 1B). Signal was normalized to the background noise so that the signal‐to‐noise ratio (SNR) could be used to quantitate signal enhancement due to Mn^2+^ uptake. In the absence of Mn^2+^, slices still have signal in T_1w_ images, therefore comparing the SNR from those slices with those slices containing Mn^2+^ enables a more complete analysis of the effect of Mn^2+^ incubation on the T_1w_ images. The SNR was computed for each ROI and the SNR of slices without Mn^2+^ was subtracted to calculate the delta SNR (ΔSNR). ΔSNR was used as a proxy for Mn^2+^ uptake in slices. The average values and standard deviation (SD) were calculated for each group; the values are listed in Table S2.

### 2.5 Cell damage measurements

Propidium iodide (PI) fluorescence was used to measure cell damage after Mn^2+^ and/or drug incubation, using 2 μM PI (Life Technologies, Carlsbad, CA, USA). Briefly, slices were washed of media containing Mn^2+^ and pharmacological drugs then placed in media containing PI for 15–20 minutes (Figure S1C). Images were captured with a fluorescent microscope modified to accommodate slices, as described below in the Electrophysiology section (2.6). PI enters damaged cells through compromised membranes; thus, increased staining is an indicator of poor cellular health. Cell damage was determined for the total slice as previously described^52^ for MRI data analysis, using Image J (NIH, Bethesda, MD, USA), and reported as % change compared with slices with no Mn^2+^. The average values and SD were calculated for each group.

### 2.6 Electrophysiology

Slices were removed from culture media then placed in a submersion‐type chamber perfused at 2 ml/min with a CSF bubbled with 95% O_2_ and 5% CO_2_. Data were amplified using Axon MultiClamp 700A (Molecular Devices, San Jose, CA, USA) then digitized, acquired and analyzed with pClamp10 software (Molecular Devices).

Schaffer collateral synapses were measured after 1 hour of MRI to assess slice health. Field‐evoked postsynaptic potentials were evoked with a concentric bipolar electrode (FHC, Bowdoin, ME, USA) in the CA3 region. The recording electrode was placed in the dendritic zone of CA1. Pulses were 0.1 ms with an interstimulus interval of 100 ms at 0.1 Hz. Sweeps at intensities ranging from 0 to 10 μA were applied to find the maximum intensity response. Intensity was adjusted to create an input/output curve with intervals every 10% from 0% to maximum response.

Whole cell experiments were recorded in the same aCSF with patch pipettes with 3–5 MΩ resistance 1 hour after Mn^2+^ exposure. Miniature excitatory postsynaptic currents (mEPSCs) were recorded with internal solution containing 130 Cs‐gluconate, 8 KCl, 1 EGTA, 10 HEPES, 4 ATP, 5 QX‐314; pH 7.4, and 285–295 mOsm. Recordings lasted 5 minutes per cell, and occurred in the presence of TTX, bicuculline (20 μM; Sigma) and APV. Cells were held in a voltage clamp at –80 mV to isolate AMPA receptor‐mediated events. Two hundred consecutive events were analyzed per cell, and the average frequency and amplitude were measured. Intrinsic excitability and spiking properties were recorded with internal solution containing (in mM) 130 (K)gluconate, 10 KCl, 0.2 EGTA, 10 HEPES, 4 (Mg)ATP, 0.5 (Na)GTP, and 10 (Na)phosphocreatine (pH 7.25, 280–290 mOsm). Resting membrane potential (Vm) and input resistance were recorded in a current clamp, and subsequent 800‐ms depolarizing current steps were injected at 20‐pA intervals to detect the rheobase values of each cell. The rheobase value is the amount of current necessary to inject into a cell to elicit one spike.

### 2.7 Statistics

Statistical analysis on MRI, PI and electrophysiological data was performed using unpaired Student's t‐test, assuming two‐tailed distribution, with statistical significance defined as *p* < 0.05. For the MRI and PI figures the values are average +/− SD, and electrophysiology is average +/− standard error of the mean (SEM); * = *p* < 0.05, ** = *p* < 0.01, and *** = *p* < 0.005.

## 3 RESULTS

### 3.1 Mn^2+^ concentration and incubation durations influence MRI SNR

We first determined the Mn^2+^ concentration and incubation times that provide increases in SNR in regions that accumulate Mn^2+^. Representative T_1w_ images of OHSCs at various concentrations of Mn^2+^ solution, 0–150 μM, and incubation times of 1–24 hours, are shown in Figure 1A. During MRI scanning, slices were maintained by continuous perfusion of warmed and bubbled aCSF (Figure S1). After 1 hour in the MRI no significant detrimental effects on OHSC viability were observed with electrophysiological measurements (Figure S2). MRI acquisition properties were used such that slices without Mn^2+^ showed low MRI signal and little contrast in the T_1w_ images at all incubation times (Figure 1A,C). At 10 and 25 μM Mn^2+^ concentrations, longer incubation times resulted in increased signal intensity and more distinct hippocampal cytoarchitecture due to shortening of T_1_ due to Mn^2+^ accumulation (Figure 1A,B). The high resolution of 50 μm in plane enabled clear demarcation of hippocampal subregions. The signal intensity and contrast increased with Mn^2+^ concentration and incubation times, enabling improved hippocampal subregion detection (Figure 1A,C). Interestingly, the ΔSNR after 24 hours with 150 μM Mn^2+^ dropped over time (Figure 1A,C). This signal drop was probably due to T_2_‐shortening effects becoming dominant at this high Mn^2+^ concentration, even at the short TEs used.

### 3.2 Higher Mn^2+^ concentration and longer incubation times decrease slice viability

**FIGURE 2:**
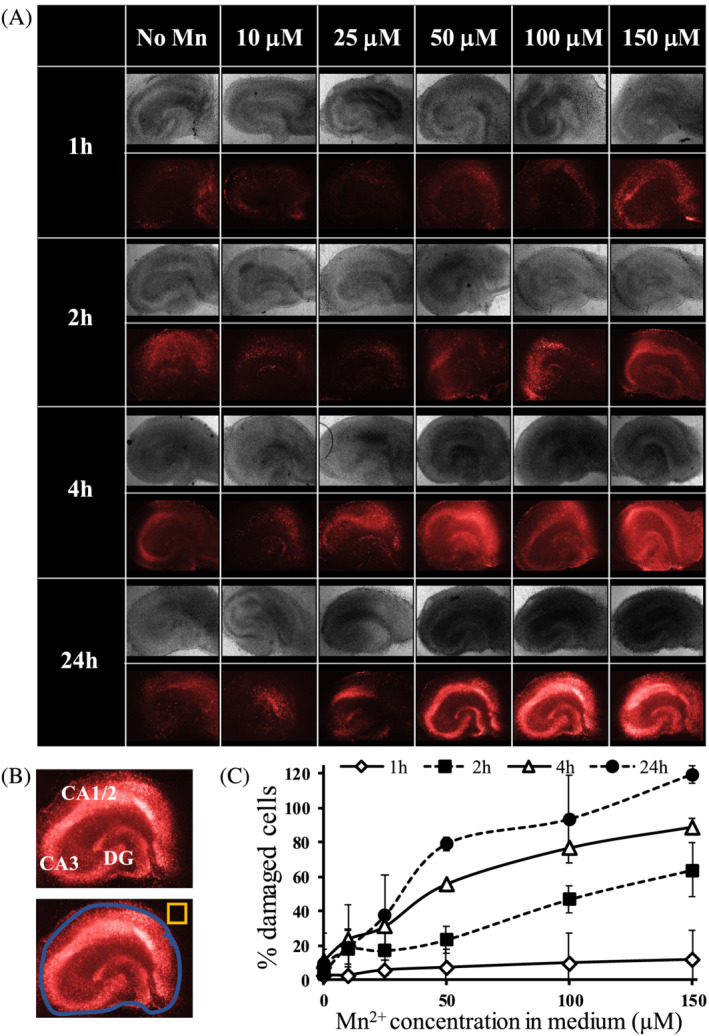
OHSC cell damage detected with propidium iodine (PI). A, Representative infrared (IR: top) and PI staining (bottom) images of OHSCs after Mn^2+^ incubation. B, Depiction of OHSC slice and background ROI selection. C, % change of damaged cells compared with slices with no manganese, for all Mn^2+^ concentrations and incubation times; n = 3–6 slices, values are average ± SD. The % of damaged cells increased with increased Mn^2+^ concentrations and incubation times

Like most heavy metals, Mn^2+^ is essential but can be cytotoxic at higher concentrations. Cell damage measurements using PI staining were performed on OHSCs for the various Mn^2+^ concentrations and incubation durations (Figure 2). PI detects cell damage by entering cells with compromised cellular and nuclear membranes and binding to DNA, with an increase in red fluorescence indicating more damaged cells.^52^ PI staining increased with higher concentrations and longer incubation times of Mn^2+^ in OHSCs (Figure 2A,C).

As a further check on the effects of Mn^2+^ on cell function, spontaneous mEPSCs were measured from OHSC neurons after 2‐hour incubations with increasing levels of Mn^2+^. There was a dose‐dependent decrease in spontaneous network activity (Figure S3).

Based on the data from these experiments, the 2‐hour incubation time at 25 μM Mn^2+^ was chosen for subsequent studies. With these parameters the cytoarchitecture of the hippocampus was visible, cell damage was low, and activity remained stable compared with longer incubation times and higher Mn^2+^ concentrations.

### 3.3 Modulation of Ca^2+^ channel activity reduces Mn^2+^ uptake

**FIGURE 3:**
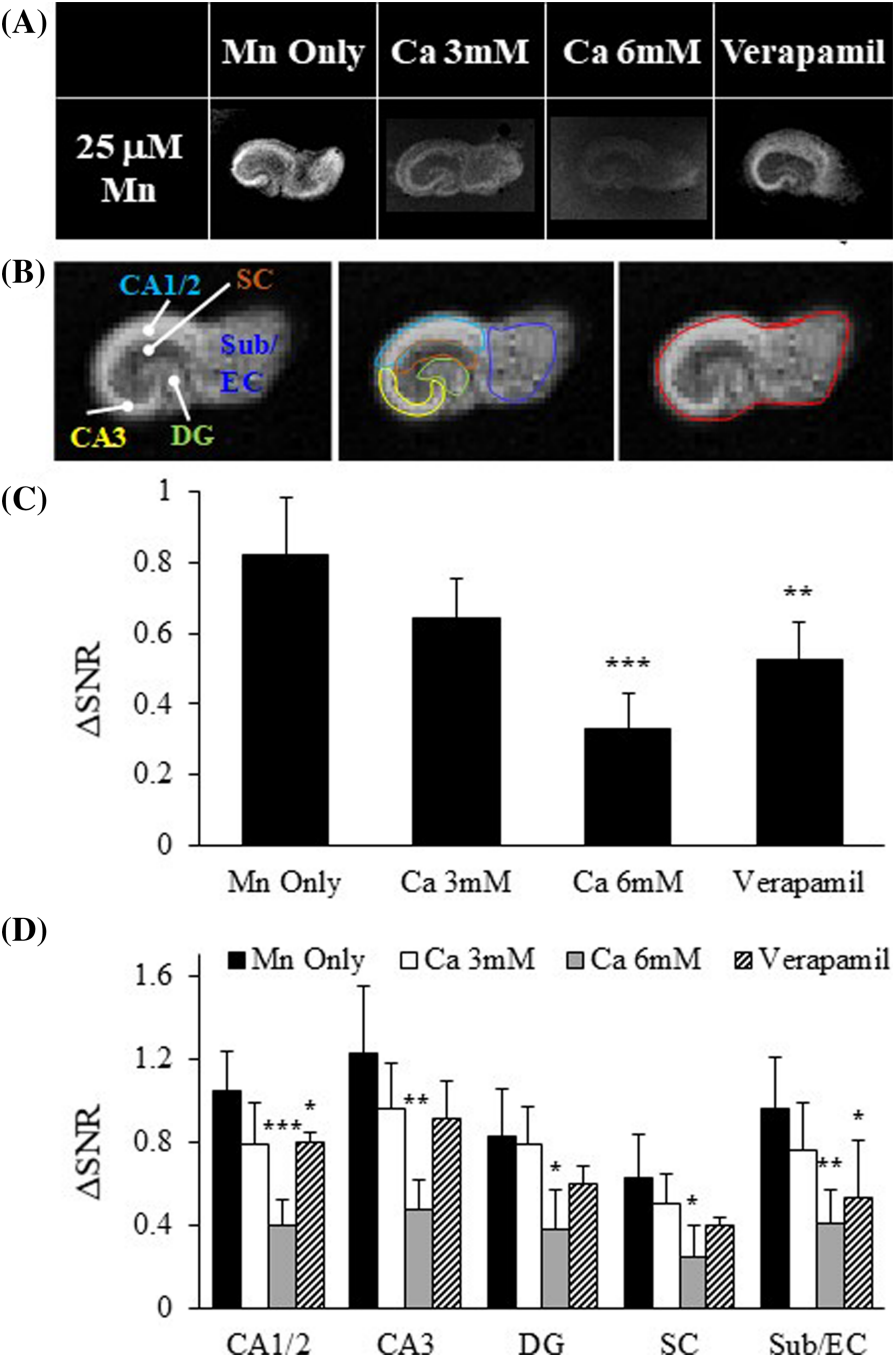
Competition for and blockade of Ca^2+^ channels reduces Mn^2+^ uptake. A, T_1_‐weighted images of OHSCs in the presence of Ca^2+^ or verapamil. B, ROIs for the slice and hippocampal subregions. C, ΔSNR bar plot for whole OHCS in the presence of Ca^2+^ channel competition or antagonism. D, ΔSNR bar plot for the subregions of the hippocampus under the same conditions as C; n = 3–6 slices, values are average ± SD. CA3 and CA1/2, Cornu Ammonis; DG, dentate gyrus; SC, Schaffer collaterals; Sub/EC, subiculum/entorhinal cortex. *p < 0.05, **p < 0.01, ***p < 0.005

Mn^2+^ transport through Ca^2+^ channels was studied with the addition of Ca^2+^ and verapamil, a Ca^2+^ channel antagonist, to the Mn^2+^‐incubated OHSCs (Figure 3). The addition of 3 and 6 mM Ca^2+^ decreased Mn^2+^ uptake in OHSCs (by 22% and 60%, respectively) (Figure 3C, Table S2). This decrease in Mn^2+^ accumulation indicates that increased extracellular Ca^2+^ is competing with Mn^2+^ for cell entry. Blocking Ca^2+^ channels with verapamil decreased Mn^2+^ uptake by 36% in the slice, similar to Ca^2+^ exposure (Figure 3A,C, Table S2). Mn^2+^ uptake can vary by hippocampal region, so subregion‐specific ROIs were selected and SNR was calculated as a measure of Mn^2+^ accumulation (Figure 3B,D, Table S2). Lower Mn^2+^ uptake was evident in all areas of hippocampus in the presence of 6 mM Ca^2+^, but the most sensitive region to competitive modulation was CA1/2, where these treatments further reduced Mn^2+^ uptake (Figure 3D, Table S2). Cell damage was slightly but not significantly reduced with the addition of Ca^2+^ compared with Mn^2+^ but increased with the addition of verapamil (Figure S4A). These results indicate that Ca^2+^ competes with Mn^2+^ to enter the cells.

### 3.4 Modulating glutamate receptor‐mediated synaptic activity impacts Mn^2+^ uptake

**FIGURE 4:**
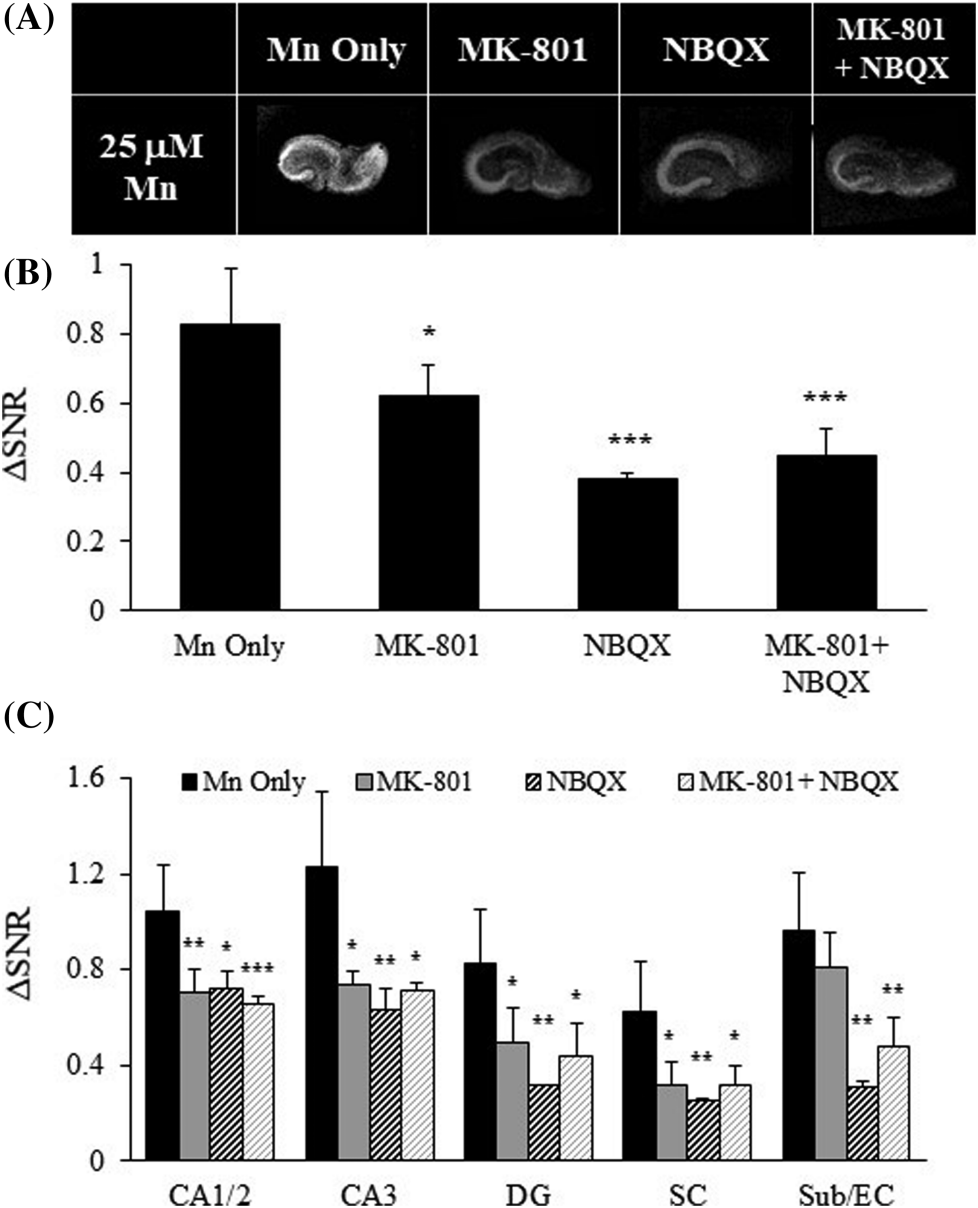
Modulating glutamate receptor mediated synaptic activity impacts Mn^2+^ uptake. A, T_1_‐weighted images of OHSCs in the presence of MK‐801 and/or NBQX. B, ΔSNR bar plot for whole OHCS. C, ΔSNR bar plots for hippocampal subregions; n = 2–6 slices and values are average ± SD. CA3 and CA1/2, Cornu Ammonis; DG, dentate gyrus; SC, Schaffer collaterals; Sub/EC, subiculum/entorhinal cortex. *p < 0.05, **p < 0.01, ***p < 0.005

Mn^2+^ is known to enter cells via Ca^2+^ channels, which are opened during periods of cellular activity. Mn^2+^ uptake was measured when slice activity was decreased with incubation of excitatory (glutamatergic) receptor antagonists. Mn^2+^ cell uptake was studied in the presence of glutamatergic receptor antagonists: MK‐801 to block NMDA receptors and NBQX to block AMPA receptors (Figure 4). As expected, blocking activity with MK‐801 and NBQX decreased Mn^2+^ cell uptake (by 24% and 54%, respectively), and the combination of both antagonists decreased Mn^2+^ uptake by 46% (Figure 4A,B, Table S2). Mn^2+^ uptake for different areas of the hippocampus was reduced by glutamatergic blockade, with all subregions being similarly affected (Figure 4C, Table S2). Modest increases in cell damage was detected in the presence of MK‐801 and NBQX (Figure S4B). These results indicate that blockade of those synaptic receptors which usually flux Ca^2+^ also reduces Mn^2+^ entry, and to an extent that is similar to increased calcium concentrations.

### 3.5 Activity blockade has varying effects on Mn^2+^ accumulation

**FIGURE 5:**
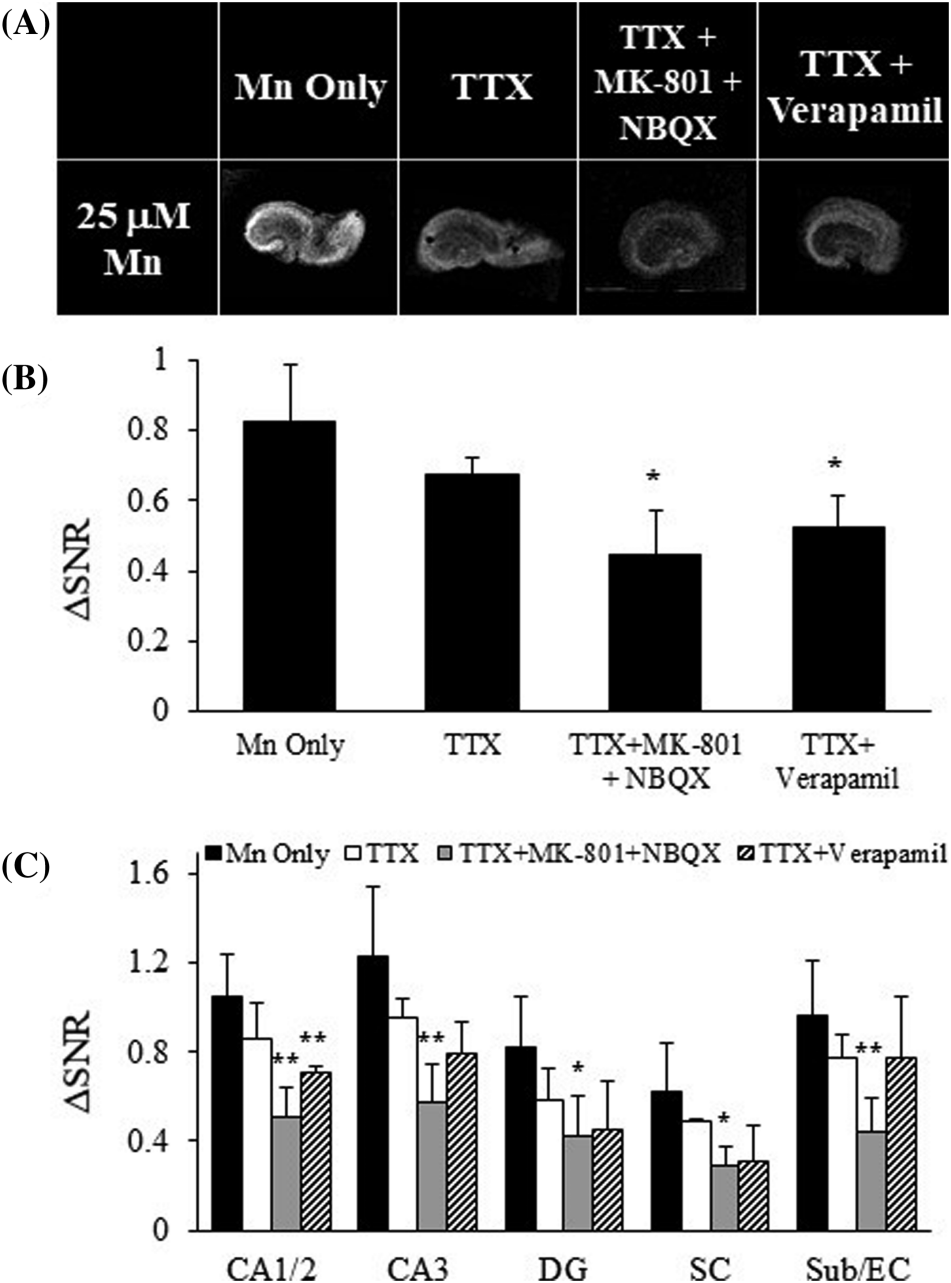
Activity blockade has varying effects on Mn^2+^ accumulation. A, T_1_‐weighted images of OHSCs in the presence of TTX. TTX was added to medium alone or with MK‐801 and NBQX, or with verapamil. B, ΔSNR bar plots for whole OHCS under different conditions. C, ΔSNR bar plots for hippocampal subregions; n = 2–6 slices and values are average ± SD. CA3 and CA1/2, Cornu Ammonis; DG, dentate gyrus; SC, Schaffer collaterals; Sub/EC, subiculum/entorhinal cortex. *p < 0.05, **p < 0.01, ***p < 0.005

Mn^2+^ uptake was impaired by the presence of competitors or antagonists for receptors that usually flux Ca^2+^. These treatments are also associated with decreased OHSC activity. AMPA/NMDA receptor antagonists block activity of these channels during synaptic activity. TTX blocks Na^+^ channels, which impairs the ability of neurons to fire action potentials, but does not block spontaneous vesicle release.^53^ The addition of TTX to the medium did not results in a significant reduction (18%) of Mn^2+^ uptake in the slice (Figure 5). Adding TTX in conjunction with MK‐801 and NBQX or verapamil did decrease Mn^2+^ uptake (by 46% and 37%, respectively), similar to reductions observed by inhibiting the AMPA/NMDA receptors or VGCCs alone (Figure 5A,B). Hippocampal subregions all demonstrated similar reductions in Mn^2+^ uptake, indicating that the slice was globally affected (Figure 5C, Table S2). PI staining was not significantly altered in the presence of TTX alone, but higher levels of cell damage were evident when TTX was combined with glutamate or Ca^2+^ receptor antagonists (Figure S4C). These data indicate that incubations with TTX alone did not significantly impact Mn^2+^ uptake but requires the addition of other drugs to achieve reduced Mn^2+^ levels. These results indicate that synaptic activity may be more effective at driving Mn^2+^ uptake than propagating transsynaptic activity in OHSCs.

### 3.6 Mn^2+^ uptake is reduced in the presence of other metal ions for cell entry

**FIGURE 6:**
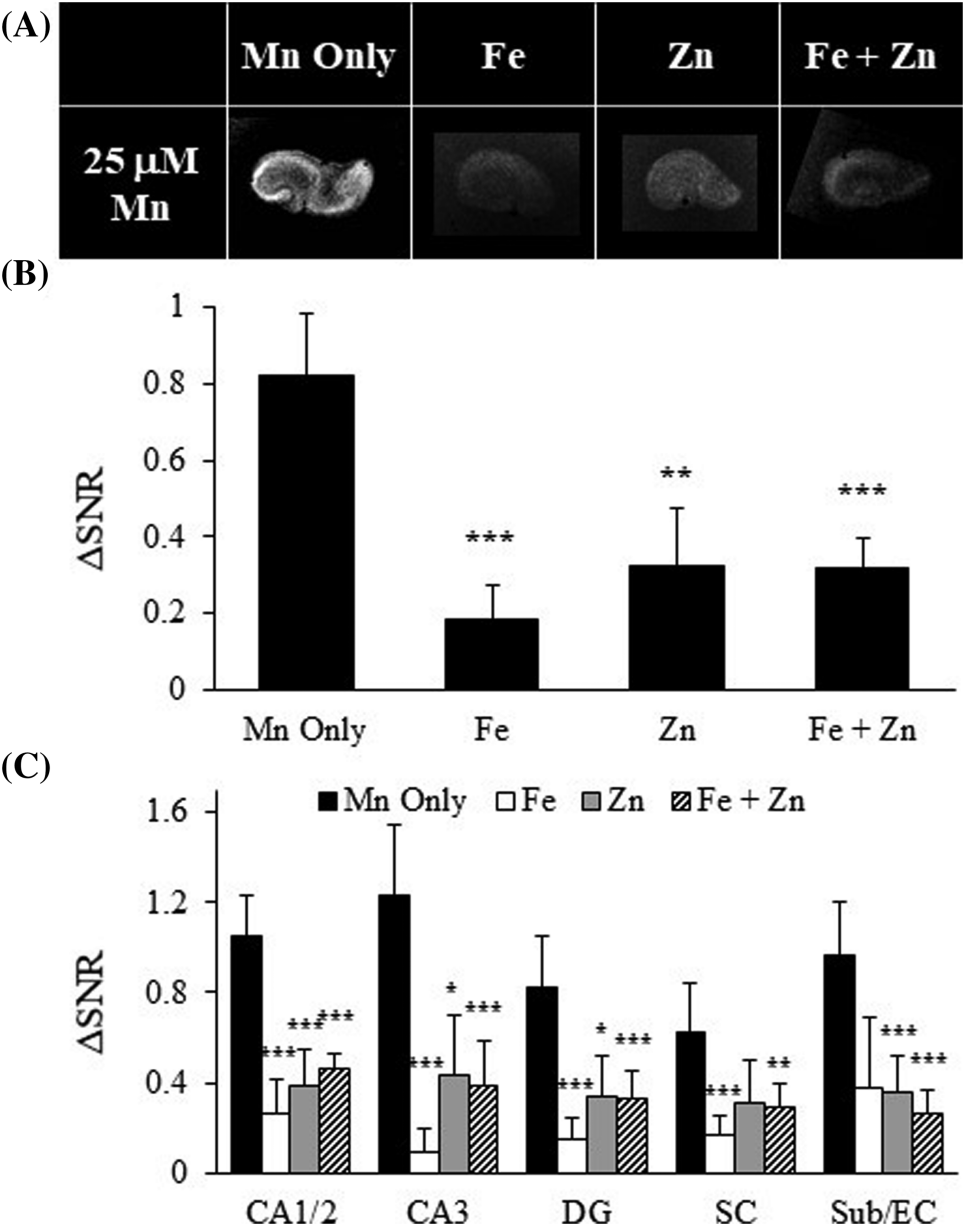
Mn^2+^ competition reduces Mn^2+^ transport through divalent metal transporters. A, T_1_‐weighted images of OHSCs in the presence of Fe^2+^ and Zn^2+^ or both. B, ΔSNR bar plots for whole OHCS. C, ΔSNR bar plot for the subregions of the hippocampus; n = 3–6 slices, values are average ± SD. CA3 and CA1/2, Cornu Ammonis; DG, dentate gyrus; SC, Schaffer collaterals; Sub/EC, subiculum/entorhinal cortex. *p < 0.05, **p < 0.01, ***p < 0.005

Mn^2+^ is known to enter cells through metal transporters, so Zn^2+^ and/or Fe^2+^ were added to the incubation media to determine if their presence affected Mn^2+^ uptake in hippocampal slices. Both metals significantly decreased Mn^2+^ uptake (Figure 6A,B, Table S2). Fe^2+^ was slightly more effective at reducing Mn^2+^ uptake than Zn^2+^, with reductions of 74% at the highest Fe^2+^ concentration and 61% at the highest Zn^2+^ concentration. When both metals were present, the decrease in Mn^2+^ uptake was similar to that of Fe^2+^ alone (58%), suggesting a possible preference for the transport of Mn^2+^ through Fe^2+^ transporters than through Zn^2+^ transporters. The presence of divalent metals reduced Mn^2+^ uptake in all subregions of the hippocampus (Figure 6C, Table S2). PI levels for individual metals were not increased compared with Mn^2+^ alone, but higher cell damage was detected in the presence of both metals simultaneously (Figure S4B). These results indicate that metals compete with Mn^2+^ transport, subregions are not preferentially affected by this competition, and that an overabundance of metals in the media can impact slice viability.

### 3.7 Results summary

**FIGURE 7:**
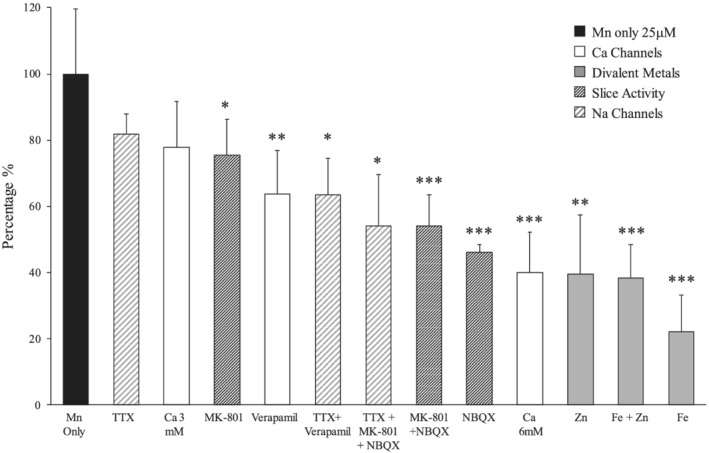
Summary of results for incubations with all ions, metals and drugs. Mn^2+^ uptake was least affected by blocking activity, followed by competition and blockade of Ca^2+^ channels, and most reduced when in competition with divalent metals. Results are presented as percentage compared with Mn^2+^ only, which was set to 100%. *p < 0.05, **p < 0.01, ***p < 0.005

A summary of the results is shown in Figure 7. Results are presented as % of Mn^2+^ uptake compared with Mn^2+^ alone, which was normalized to 100%. The least effective treatment for blocking Mn^2+^ uptake was TTX, while modulation of Ca^2+^ channels or glutamatergic receptors are more effective. Divalent metals were most effective at blocking Mn^2+^ uptake, likely due to competition for metal transporters.

## 4 DISCUSSION

The goal of this study was to determine how much Mn^2+^ enters cells through a variety of uptake routes. First, a protocol was established to perform MRI on living OHSCs to detect the optimal Mn^2+^ concentration and incubation time to achieve maximal SNR with minimal impact on slice viability (Figures 1 and 2). High‐resolution MRI at about 50 μm isotropic resolution could be readily obtained, which likely makes these slices useful for other aspects of understanding the origin of MRI contrast.^54^, ^55^, ^56^ Although T_1w_ mapping is only semiquantitative, the goal of these experiments was to obtain high‐resolution scans with short scan times. The changes in SNR between treatments enabled an ordering of the magnitude of the treatments' effects on the images, thus, for the purpose of these experiments, it is likely a sufficient method to determine a hierarchy of those agents which influence Mn^2+^ uptake. Second, it was demonstrated that the presence of divalent cations such as Ca^2+^ and reducing slice activity decreased Mn^2+^ entry (Figures 3, 4, 5). The presence of metals such as Zn^2+^ and Fe^2+^ compete to further reduce Mn^2+^ uptake (Figure 6). Overall, the presence of metals Zn^2+^ and Fe^2+^ had the largest effect on Mn^2+^ uptake, while the reduction of slice activity and modulation of Ca^2+^ channels reduced impact on Mn^2+^ uptake (Figure 7). It is important to note that the experiments were performed after 1 hour incubation at 25 μM Mn^2+^. These conditions may favor metal ion transporters while shorter incubations and lower concentrations may prefer activity‐dependent uptake.^6^, ^57^ Future studies should explore the effects of timing and lower concentrations of Mn^2+^.

Neurons depend on tight regulation of Ca^2+^ by neurons and glia.^58^ Ca^2+^ and other ions are used to create charge and concentration gradients across the membrane for effective synaptic transmission.^59^ Neurons maintain these gradients with Ca^2+^ channels and exchangers, some of which are known to also flux Mn^2+^. Mn^2+^ behaves as a Ca^2+^ analog due to its similar radius and charge,^9^, ^10^ where it can flow through VGCC,^10^, ^46^ Ca^2+^/Na^+^ exchangers,^12^ active Ca^2+^ uniporters^13^ and Na^+^/Mg^2+^ antiporters.^60^ Here, we have demonstrated that the presence of Ca^2+^ interferes with Mn^2+^ uptake in a dose‐dependent manner, and that blockade of VGCCs leads to decreased Mn^2+^ accumulation in slice (Figure 3). These data indicate that Ca^2+^ competes with Mn^2+^ for cell entry likely due to their sharing the same transporters.

Performing high‐resolution MRI on slice culture increases the capacity to detect changes in Mn^2+^ uptake in different subregions of the hippocampus. Mn^2+^ uptake in CA1/2 was reduced when coincubated with increased Ca^2+^ or Ca^2+^ channel blockers (Figure 3). CA1/2 is particularly susceptible to Ca^2+^‐mediated excitotoxicity after global ischemia,^61^ which may indicate that CA1/2 is able to take up more divalent ions like Ca^2+^ and/or Mn^2+^. In addition, CA1 has been shown to take up more Mn^2+^ than CA3,^62^ which may increase the dynamic range of Mn^2+^ uptake and blockade capacity for this hippocampal region. Regardless of the region‐specific mechanism, it is clear that the presence of Ca^2+^ or Ca^2+^ channel blockers reduce Mn^2+^ uptake in OHSCs, likely due to a competition mechanism.

Increased Mn^2+^ is taken up and transported between neurons experiencing high activity in vivo and in vitro.^2^, ^5^, ^6^, ^28^, ^63^, ^64^ Mn^2+^ likely follows Ca^2+^ flux through VGCCs^10^, ^43^ and excitatory glutamatergic receptors like AMPA and NMDA,^44^, ^65^, ^66^ which both flux Ca^2+^ (and presumably Mn^2+^) when activated.^67^, ^68^ Our studies support these findings by demonstrating that blocking NMDA and AMPA receptors reduces Mn^2+^ accumulation (Figure 4). Propagating activity was inhibited by incubating slices with TTX, which blocks Na^+^ channels and action potentials.^69^ TTX did not significantly reduce Mn^2+^ uptake, but when AMPA/NMDA receptors and VGCCs were also blocked, less Mn^2+^ accumulated in slices (Figure 5). These results indicate that synaptic activity mediated by AMPA/NMDA receptors or VGCCs play a more important role in Mn^2+^ uptake in our slices. Beyond altering neuronal activity, TTX incubation can also increase astrocytic Ca^2+^ influx,^70^ thus TTX may increase Mn^2+^ accumulation in the astrocytes of these slices to complement Mn^2+^ neuronal entry. The combination of TTX with other synaptic receptor antagonists did reduce Mn^2+^ accumulation, indicating that more complete synaptic activity blockade does inhibit Mn^2+^ entry.

The developing and adult brain require precise levels of essential metals to function properly. In neonatal rodents, high Mn^2+^ is observed in blood and brain tissue, perhaps in response to a greater need during development.^39^, ^71^ DMT1, transferrin and ZIP8 are all transporters that can transport metal ions such as Fe^2+^, Zn^2+^, Cu^2+^, Co^2+^ and Mn^2+^ across membranes to maintain metal ion homeostasis.^15^, ^19^, ^72^, ^73^ Here, we have demonstrated that the presence of metal ions like Fe^2+^ and Zn^2+^ decrease Mn^2+^ uptake in OHSCs (Figure 6). Mn^2+^ likely competes with other metals for access to and transport across metal transporters. This is in agreement with other researchers who have demonstrated that the presence of Mn^2+^ decreases Fe^2+^ levels.^40^, ^74^ Variability between hippocampal subregions was detected with this high field MRI preparation, where CA1/2 and CA3 took up more Mn^2+^ than DG, SC and Sub/EC. CA1/2 and CA3 were also the most sensitive to competitors, indicating that the reduction was more pronounced in these regions. This is likely due to varying distributions of VGCCs, calcium permeable AMPA or NMDA receptors, or metal transporters. For example, at this early postnatal age, more VGCCs are present in CA1/2 and CA3,^75^ and these could produce the increased Mn^2+^ uptake in these regions. NMDA receptors that flux Ca^2+^ and Mn^2+^ are more prevalent in CA1 and DG but not in CA3 at this age,^76^ which may explain the increased Mn^2+^ uptake in CA1, but is in conflict with the observed Mn^2+^ uptake patterns in CA3 and DG. Because metal transporters demonstrated the largest competition impact on Mn^2+^ uptake, the regional presence of metals and their transporters are likely the most important influence on Mn^2+^ uptake in our experiments. Metals demonstrate a varied distribution, with CA1 containing the most Fe^2+^ and CA3 containing more Zn^2+^.^77^ Although the presence of metals does not necessarily mean more transporters are available, it could provide an indication regarding the levels of metal ions fluxing in different hippocampal subregions. More DMT1 is present in CA3 than in CA1, which may bring more Mn^2+^ and other metal ions into CA3^78^ and may explain the higher levels of Mn^2+^ in the CA3 of young rats.^79^ There is conflicting research regarding location specificity of Mn^2+^ accumulation in the hippocampus, with some work reporting more Mn^2+^ in dissociated CA1 neurons,^80^ while other work reports more in CA3 and DG in vivo.^79^ Regional variances in Mn^2+^ uptake may provide an insight into how the areal differences in levels of VGCCs, Ca^2+^‐permeable glutamatergic receptors and metal transporters influence the hippocampus's varying sensitivities to injury. For example, CA3 is more sensitive to seizure while CA1 is more sensitive to ischemia and neurodegeneration.^3^, ^61^ After seizure Mn^2+^ is known to accumulate in CA3 and in DG due to increased activity and mossy fiber sprouting, not neurodegeneration or edema^81^; however, this Mn^2+^ accumulation has not been widely observed.^82^ This work demonstrates the elegant complexity of both the hippocampal system and Mn^2+^ uptake mechanisms.

Previous in vivo studies describing Mn^2+^ uptake in the brain use either systemic or intracerebral injection and subsequent imaging, either with MRI or PET and radioactive isotopes of Mn^2+^.^4^, ^25^, ^62^ in vitro studies have also quantified Mn^2+^ accumulation in hippocampal slices using synchotron x‐ray fluorescence.^40^ While these studies have worked to identify the mechanism of Mn^2+^ entry in the context of competition with metals, divalent cations or activity modulation, this study provides a unique examination of Mn^2+^ entry combining all three variables. These results allow for an estimate of which transport mechanism Mn^2+^ preferentially uses. Under the conditions in this study, Fe^2+^ and Zn^2+^ compete the most with Mn^2+^ for uptake into the OHSCs, indicating that metal transporters are the primary drivers for Mn^2+^ accumulation. Calcium influx mechanisms related to activity also contribute to a large amount of Mn^2+^ uptake, albeit less than those related to metal transport mechanisms. These studies establish the use of living tissue preparations for studying the mechanisms of Mn^2+^ uptake. This work should be readily extendable to methods such as human brain organoids, thus enabling the effects of diseases mimicked in the dish to help interpret contrast in MEMRI.

## Supporting information


**Figure S1:** Experimental Setup with the OHSC MRI perfusion chamber (**A**). Slices were removed from the cell culture insert. **B**. The membrane insert was cut and the slice was rinsed in aCSF, placed in a perfusion chamber and fixed with a plastic anchor. To keep cultures alive during imaging, slices were continuously perfused with the aCSF at 0.9 μL/min, bubbled with 95% O_2_ and 5% CO_2_ at temperature of 37.5°C (DG ‐ dentate gyrus, CA3 and CA1/2 ‐ Cornu Ammonis, SC ‐ Schaffer collaterals, Sub/EC ‐ subiculum/entorhinal cortex. **C**. Schematic of drug application, incubation and imaging timepoints.
**Figure S2:** Validation of slice viability after 1 hour in the MRI. **A**. Membrane properties and intrinsic excitability are not affected by 1 h in MRI. **B**. Representative traces and graph depicting spiking behavior of CA3 principal neurons, data used to determine Rheobase (minimum amount of current injection needed to cause one spike). **C**. Representative traces and Input/Output curves of fEPSP (field excitatory post synaptic potential) evoked by field stimulation and recording along the Shaffer collaterals. Values are average ± SEM.
**Figure S3:** Spontaneous slice activity is reduced after 2 hours Mn^2+^ exposure in a dose dependent manner. **A.** Representative traces of mEPSC (miniature excitatory postsynaptic current) measurements at different Mn^2+^ concentrations. **B**. Average mEPSC frequency is inversely proportional to Mn^2+^ concentration, while mEPSC amplitude is unaffected. Constant amplitude measurements indicate that postsynaptic events occur normally, but reduced frequency indicates impaired presynaptic vesicle release. 9–12 cells, 3–4 slices per group, values are average ± SEM.
**Figure S4**: OHSC cell damage measurements with propodium iodine (PI) in the presence of treatments. OHSC slice outlines were detected with infrared (IR) images. **A**. Top: PI staining and IR images of OHSC in the presence of Ca^2+^ or verapamil, bottom: corresponding % change of PI staining compared to slices with no Mn^2+^. **B**. Top: PI staining and IR images of OHSC in the presence of glutamate receptor antagonists, bottom: corresponding % change of PI staining compared to slices with no Mn^2+^. **C**: Top: PI staining and IR images of OHSC in the presence of activity blockade by TTX alone, or in the presence of glutamatergic or Ca^2+^ channel antagonists, bottom: corresponding % change of PI staining compared to slices with no Mn^2+^. **D**. Top: PI staining and IR images of OHSC in the presence of Fe^2+^ and Zn^2+^ or both, bottom: corresponding % change of PI staining compared to slices with no Mn^2+^. Values are average ± SD.
**Figure S5**: The ferromagnetic contrast of Fe^2+^ in MRI images. Mn^2+^ cell uptake was studied in the presence of Fe^2+^ and Zn^2+^ or both. To account for the ferromagnetic contrast generated by Fe^2+^, OHSC were incubated separately with 1mM Fe^2+^ only. A. T_1_ weighted images of OHSC after 2 hours incubation at 1mM Fe^2+^ only and at 25mM Mn^2+^ (only and) with the addition of Fe^2+^, Zn^2+^ or both. B. DSNR graph for the OHSC under different conditions for 25mM Mn^2+^ concentration. For each condition n = 3–6 slices and values are average ± SD. (*p < 0.05, **p < 0.01, ***p < 0.005).
**Table S1**. A summary of all metals and drugs used in the study and the incubation times.
**Table S2**. A summary of average values (+/− standard deviation) and Student's T‐Test p values for SNR values for whole slice and hippocampal subregions.

Supporting info item.

## Data Availability

The data that support the findings of this study are available from the corresponding author upon reasonable request.
