# Supplementary material for: A hierarchy of manganese competition and entry in organotypic hippocampal slice cultures
Source: NMR Biomed. 2021 Feb 3;34(4):e4476. doi: 10.1002/nbm.4476 (PMC7988546; doi:10.1002/nbm.4476)
Supplement: Supplementary file 1 — Figure S1: Experimental Setup with the OHSC MRI perfusion chamber (A). Slices were removed from the cell culture insert. B. The membrane insert was cut and the slice was rinsed in aCSF, placed in a perfusion chamber and fixed with a plastic anchor. To keep cultures alive during imaging, slices were continuously perfused with the aCSF at 0.9 μL/min, bubbled with 95% O2 and 5% CO2 at temperature of 37.5°C (DG ‐ dentate gyrus, CA3 and CA1/2 ‐ Cornu Ammonis, SC ‐ Schaffer collaterals, Sub/EC ‐ subiculum/entorhinal cortex. C. Schematic of drug application, incubation and imaging timepoints. Figure S2: Validation of slice viability after 1 hour in the MRI. A. Membrane properties and intrinsic excitability are not affected by 1 h in MRI. B. Representative traces and graph depicting spiking behavior of CA3 principal neurons, data used to determine Rheobase (minimum amount of current injection needed to cause one spike). C. Representative traces and Input/Output curves of fEPSP (field excitatory post synaptic potential) evoked by field stimulation and recording along the Shaffer collaterals. Values are average ± SEM. Figure S3: Spontaneous slice activity is reduced after 2 hours Mn2+ exposure in a dose dependent manner. A. Representative traces of mEPSC (miniature excitatory postsynaptic current) measurements at different Mn2+ concentrations. B. Average mEPSC frequency is inversely proportional to Mn2+ concentration, while mEPSC amplitude is unaffected. Constant amplitude measurements indicate that postsynaptic events occur normally, but reduced frequency indicates impaired presynaptic vesicle release. 9–12 cells, 3–4 slices per group, values are average ± SEM. Figure S4: OHSC cell damage measurements with propodium iodine (PI) in the presence of treatments. OHSC slice outlines were detected with infrared (IR) images. A. Top: PI staining and IR images of OHSC in the presence of Ca2+ or verapamil, bottom: corresponding % change of PI staining compared to slices with no Mn2+. [file NBM-34-e4476-s001.docx]

**Supplementary Material**

**Supplemental Figure Legends**

**Figure S1:** Experimental Setup with the OHSC MRI perfusion chamber (**A**). Slices were removed from the cell culture insert. **B**. The membrane insert was cut and the slice was rinsed in aCSF, placed in a perfusion chamber and fixed with a plastic anchor. To keep cultures alive during imaging, slices were continuously perfused with the aCSF at 0.9 µL/min, bubbled with 95% O_2_ and 5% CO_2_ at temperature of 37.5^o^C (DG - dentate gyrus, CA3 and CA1/2 - Cornu Ammonis, SC - Schaffer collaterals, Sub/EC - subiculum/entorhinal cortex. **C**. Schematic of drug application, incubation and imaging timepoints.

**Figure S2:** Validation of slice viability after 1 hour in the MRI. **A**. Membrane properties and intrinsic excitability are not affected by 1 h in MRI. **B**. Representative traces and graph depicting spiking behavior of CA3 principal neurons, data used to determine Rheobase (minimum amount of current injection needed to cause one spike). **C**. Representative traces and Input/Output curves of fEPSP (field excitatory post synaptic potential) evoked by field stimulation and recording along the Shaffer collaterals. Values are average ± SEM.

**Figure S3:** Spontaneous slice activity is reduced after 2 hours Mn^2+^ exposure in a dose dependent manner. **A.** Representative traces of mEPSC (miniature excitatory postsynaptic current) measurements at different Mn^2+^ concentrations. **B**. Average mEPSC frequency is inversely proportional to Mn^2+^ concentration, while mEPSC amplitude is unaffected. Constant amplitude measurements indicate that postsynaptic events occur normally, but reduced frequency indicates impaired presynaptic vesicle release. 9-12 cells, 3-4 slices per group, values are average ± SEM.

**Figure S4**: OHSC cell damage measurements with propodium iodine (PI) in the presence of treatments. OHSC slice outlines were detected with infrared (IR) images. **A**. Top: PI staining and IR images of OHSC in the presence of Ca^2+^ or verapamil, bottom: corresponding % change of PI staining compared to slices with no Mn^2+^. **B**. Top: PI staining and IR images of OHSC in the presence of glutamate receptor antagonists, bottom: corresponding % change of PI staining compared to slices with no Mn^2+^. **C**: Top: PI staining and IR images of OHSC in the presence of activity blockade by TTX alone, or in the presence of glutamatergic or Ca^2+^ channel antagonists, bottom: corresponding % change of PI staining compared to slices with no Mn^2+^. **D**. Top: PI staining and IR images of OHSC in the presence of Fe^2+^ and Zn^2+^ or both, bottom: corresponding % change of PI staining compared to slices with no Mn^2+^. Values are average ± SD.

**Figure S5**: The ferromagnetic contrast of Fe^2+^ in MRI images. Mn^2+^ cell uptake was studied in the presence of Fe^2+^ and Zn^2+^ or both. To account for the ferromagnetic contrast generated by Fe^2+^, OHSC were incubated separately with 1mM Fe^2+^ only. A. T_1_ weighted images of OHSC after 2 hours incubation at 1mM Fe^2+^ only and at 25 mM Mn^2+^ (only and) with the addition of Fe^2+^, Zn^2+^ or both. B. DSNR graph for the OHSC under different conditions for 25 mM Mn^2+^ concentration. For each condition n=3-6 slices and values are average ± SD. (*p<0.05, **p<0.01, ***p<0.005).

**Supplemental Tables**

**Table S1**. A summary of all metals and drugs used in the study and the incubation times.

| Experiment | Metal/Drug | Concentration | Incubation time |
| --- | --- | --- | --- |
| Manganese only | Manganese (Mn^2+^) | 0 -150 µM | 1 - 24 h |
| Manganese only | Mn^2+^ | 25 µM | 2 h |
|  |  | 150 µM | 2 h |
| Calcium channels | Calcium (Ca^2+^) | 3 mM | 2 h w/Mn^2+^ |
|  |  | 6 mM | 2 h w/Mn^2+^ |
|  | Verapamil | 40 µM | 4 h (2 h + 2 h w/Mn^2+^) |
| Glutamate | NBQX (AMPA) | 1 µM | 4 h (2 h + 2 h w/Mn^2+^) |
| receptors | MK-801 (NMDA) | 1 µM | 4 h (2 h + 2 h w/Mn^2+^) |
|  |  |  |  |
| Sodium Channels | TTX | 1 µM | 2 h w/Mn |
|  | NBQX + TTX | 1 µM each | 4 h + 2 h w/Mn^2+^ |
|  | MK-801 + NBQX +TTX | 1 µM each | 4 h + 2 h w/Mn^2+^ |
|  | Verapamil + TTX | 40 µM + 1 µM | 4 h + 2 h w/Mn^2+^ |
| Divalent metal | Zinc (Zn^2+^) | 1 mM | 2 h w/Mn^2+^ |
| transporters | Iron (Fe^2+^) | 1 mM | 2 h w/Mn^2+^ |
|  | Zn^2+^ + Fe^2+^ | 1 mM each | 2 h w/Mn^2+^ |
|  | Fe^2+^ | 1 mM | 2 h |

**Table S2**. A summary of average values (+/- standard deviation) and Student’s T-Test p values for SNR values for whole slice and hippocampal subregions.

| **Condition Whole Slice** | **SNR Average ± SD** | **ΔSNR Average ± SD** | **% Reduction ΔSNR from Mn^2+^ alone** | **TTEST P-Value (versus Mn^2+^ alone)** |
| --- | --- | --- | --- | --- |
| Mn^2+^ alone | 1.85 ± 0.16 | 0.82 ± 0.16 |  |  |
| Ca^2+^ 3mM | 1.67 ± 0.11 | 0.64 ± 0.11 | 22% | 0.0691 |
| Ca^2+^ 6mM | 1.36 ± 0.10 | 0.33 ± 0.10 | 60% | 0.0011 |
| Verapamil | 1.55 ± 0.11 | 0.52 ± 0.11 | 36% | 0.0080 |
| MK-801 | 1.65 ± 0.09 | 0.62 ± 0.09 | 24% | 0.0306 |
| NBQX | 1.41 ± 0.02 | 0.38 ± 0.02 | 54% | 0.0009 |
| MK-801 + NBQX | 1.47 ± 0.08 | 0.45 ± 0.08 | 46% | 0.0021 |
| TTX | 1.70 ± 0.05 | 0.67 ± 0.05 | 18% | 0.0796 |
| TTX + MK-801 + NBQX | 1.47 ± 0.08 | 0.45 ± 0.13 | 46% | 0.0118 |
| TTX + Verapamil | 1.55 ± 0.09 | 0.52 ± 0.09 | 37% | 0.0380 |
| Fe^2+^ | 0.18 ± 0.09 | 0.21 ± 0.09 | 74% | 0.0002 |
| Zn^2+^ | 1.35 ± 0.15 | 0.33 ± 0.15 | 61% | 0.0076 |
| Zn^2+^ + Fe^2+^ | 0.32 ± 0.08 | 0.34 ± 0.08 | 58% | 0.0003 |
| **Mn2+ alone + subregions** | **SNR Average ± SD** | **ΔSNR Average ± SD** | **% Reduction ΔSNR from Mn^2+^ alone** | **TTEST P-Value (versus Mn^2+^ alone)** |
| CA1/2 | 2.08 ± 0.19 | 1.04 ± 0.19 |  |  |
| CA3 | 2.26 ± 0.32 | 1.23 ± 0.32 |  |  |
| DG | 1.85 ± 0.23 | 0.82 ± 0.23 |  |  |
| SC | 1.65 ± 0.22 | 0.62 ± 0.22 |  |  |
| Sub/EC | 1.99 ± 0.24 | 0.96 ± 0.24 |  |  |
| **Ca^2+^ 3mM + subregions** | **SNR Average ± SD** | **ΔSNR Average ± SD** | **% Reduction ΔSNR from Mn^2+^ alone** | **TTEST P-Value (versus Mn^2+^ alone)** |
| CA1/2 | 1.82 ± 0.20 | 0.79 ± 0.20 | 25% | 0.0802 |
| CA3 | 1.99 ± 0.22 | 0.96 ± 0.22 | 22% | 0.1488 |
| DG | 1.82 ± 0.18 | 0.79 ± 0.18 | 4% | 0.7994 |
| SC | 1.54 ± 0.14 | 0.51 ± 0.14 | 19% | 0.3304 |
| Sub/EC | 1.80 ± 0.22 | 0.77 ± 0.22 | 21% | 0.2251 |
| **Ca^2+^ 6mM + subregions** | **SNR Average ± SD** | **ΔSNR Average ± SD** | **% Reduction ΔSNR from Mn^2+^ alone** | **TTEST P-Value (versus Mn^2+^ alone)** |
| CA1/2 | 1.43 ± 0.20 | 0.40 ± 0.12 | 62% | 0.0008 |
| CA3 | 1.50 ± 0.14 | 0.50 ± 0.14 | 61% | 0.0017 |
| DG | 1.41 ± 0.18 | 0.39 ± 0.19 | 53% | 0.0267 |
| SC | 1.28 ± 0.14 | 0.25 ± 0.15 | 60% | 0.0238 |
| Sub/EC | 1.44 ± 0.16 | 0.41 ± 0.17 | 58% | 0.0070 |
| **Verapamil + subregions** | **SNR Average ± SD** | **ΔSNR Average ± SD** | **% Reduction ΔSNR from Mn^2+^ alone** | **TTEST P-Value (versus Mn^2+^ alone)** |
| CA1/2 | 1.83 ± 0.18 | 0.80 ± 0.27 | 24% | 0.0207 |
| CA3 | 1.95 ± 0.27 | 0.92 ± 0.05 | 25% | 0.0829 |
| DG | 1.63 ± 0.04 | 0.60 ± 0.04 | 27% | 0.0664 |
| SC | 1.43 ± 0.09 | 0.40 ± 0.09 | 36% | 0.0512 |
| Sub/EC | 1.57 ± 0.05 | 0.53 ± 0.18 | 45% | 0.0429 |

| **MK-801 + subregions** | **SNR Average ± SD** | **ΔSNR Average ± SD** | **% Reduction ΔSNR from Mn^2+^ alone** | **TTEST P-Value (versus Mn^2+^ alone)** |
| --- | --- | --- | --- | --- |
| CA1/2 | 1.74 ± 0.10 | 0.71 ± 0.01 | 33% | 0.0051 |
| CA3 | 1.77 ± 0.06 | 0.74 ± 0.06 | 40% | 0.0118 |
| DG | 1.53 ± 0.14 | 0.50 ± 0.14 | 39% | 0.0193 |
| SC | 1.35 ± 0.09 | 0.32 ± 0.10 | 48% | 0.0169 |
| Sub/EC | 1.84 ± 0.14 | 0.81 ± 0.14 | 16% | 0.2328 |
| **NBQX + subregions** | **SNR Average ± SD** | **ΔSNR Average ± SD** | **% Reduction ΔSNR from Mn^2+^ alone** | **TTEST P-Value (versus Mn^2+^ alone)** |
| CA1/2 | 1.75 ± 0.10 | 0.72 ± 0.07 | 31% | 0.0142 |
| CA3 | 1.66 ± 0.09 | 0.63 ± 0.09 | 49% | 0.0061 |
| DG | 1.35 ± 0.00 | 0.32 ± 0.001 | 61% | 0.0029 |
| SC | 1.28 ± 0.01 | 0.25 ± 0.01 | 60% | 0.0079 |
| Sub/EC | 1.34 ± 0.02 | 0.31 ± 0.02 | 68% | 0.0011 |
| **MK-801+NBQX + subregions** | **SNR Average ± SD** | **ΔSNR Average ± SD** | **% Reduction ΔSNR from Mn^2+^ alone** | **TTEST P-Value (versus Mn^2+^ alone)** |
| CA1/2 | 1.68 ± 0.04 | 0.65 ± 0.04 | 38% | 0.0030 |
| CA3 | 1.74 ± 0.03 | 0.71 ± 0.03 | 42% | 0.0100 |
| DG | 1.47 ± 0.14 | 0.44 ± 0.14 | 47% | 0.0188 |
| SC | 1.35 ± 0.08 | 0.32 ± 0.08 | 49% | 0.0188 |
| Sub/EC | 1.51 ± 0.12 | 0.48 ± 0.12 | 59% | 0.0053 |
| **TTX + subregions** | **SNR Average ± SD** | **ΔSNR Average ± SD** | **% Reduction ΔSNR from Mn^2+^ alone** | **TTEST P-Value (versus Mn^2+^ alone)** |
| CA1/2 | 1.89 ± 0.16 | 0.87 ± 0.16 | 17% | 0.1898 |
| CA3 | 1.99 ± 0.08 | 0.96 ± 0.09 | 22% | 0.0947 |
| DG | 1.62 ± 0.14 | 0.59 ± 0.0.14 | 28% | 0.1060 |
| SC | 1.52 ± 0.15 | 0.49 ± 0.01 | 22% | 0.1786 |
| Sub/EC | 1.80 ± 0.11 | 0.77 ± 0.11 | 20% | 0.1493 |
| **TTX + MK-801 + NBQX + subregions** | **SNR Average ± SD** | **ΔSNR Average ± SD** | **% Reduction ΔSNR from Mn^2+^ alone** | **TTEST P-Value (versus Mn^2+^ alone)** |
| CA1/2 | 1.54 ± 0.14 | 0.51 ± 0.14 | 52% | 0.0032 |
| CA3 | 1.60 ± 0.18 | 0.57 ± 0.18 | 54% | 0.0058 |
| DG | 1.45 ± 0.18 | 0.42 ± 0.19 | 49% | 0.0358 |
| SC | 1.32 ± 0.09 | 0.29 ± 0.09 | 54% | 0.0136 |
| Sub/EC | 1.50 ± 0.16 | 0.44 ± 0.16 | 54% | 0.0076 |
| **TTX + Verapamil + subregions** | **SNR Average ± SD** | **ΔSNR Average ± SD** | **% Reduction ΔSNR from Mn^2+^ alone** | **TTEST P-Value (versus Mn^2+^ alone)** |
| CA1/2 | 1.73 ± 0.04 | 0.70 ± 0.04 | 33% | 0.0059 |
| CA3 | 1.83 ± 0.15 | 0.14 ± 0.15 | 35% | 0.0522 |
| DG | 1.48 ± 0.22 | 0.45 ± 0.22 | 45% | 0.1852 |
| SC | 1.34 ± 0.16 | 0.31 ± 0.16 | 50% | 0.1458 |
| Sub/EC | 1.81 ± 0.27 | 0.78 ± 0.27 | 19% | 0.4954 |

| **Fe^2+^ + subregions** | **SNR Average ± SD** | **ΔSNR Average ± SD** | **% Reduction ΔSNR from Mn^2+^ alone** | **TTEST P-Value (versus Mn^2+^ alone)** |
| --- | --- | --- | --- | --- |
| CA1/2 | 0.27 ± 0.15 | 0.30 ± 0.15 | 72% | 0.0012 |
| CA3 | 0.09 ± 0.11 | 0.21 ± 0.11 | 83% | 0.0002 |
| DG | 0.15 ± 0.10 | 0.18 ± 0.10 | 78% | 0.0006 |
| SC | 0.16 ± 0.09 | 0.15 ± 0.09 | 75% | 0.0025 |
| Sub/EC | 0.38 ± 0.31 | 0.40 ± 0.31 | 58% | 0.0640 |
| **Zn^2+^ + subregions** | **SNR Average ± SD** | **ΔSNR Average ± SD** | **% Reduction ΔSNR from Mn^2+^ alone** | **TTEST P-Value (versus Mn^2+^ alone)** |
| CA1/2 | 1.41 ± 0.16 | 0.39 ± 0.15 | 63% | 0.0032 |
| CA3 | 1.47 ± 0.26 | 0.44 ± 0.26 | 64% | 0.0106 |
| DG | 1.36 ± 0.19 | 0.33 ± 0.19 | 59% | 0.0190 |
| SC | 1.34 ± 0.19 | 0.31 ± 0.19 | 51% | 0.0810 |
| Sub/EC | 1.39 ± 0.15 | 0.36 ± 0.15 | 62% | 0.0036 |
| **Zn^2+^ + Fe^2+^ + subregions** | **SNR Average ± SD** | **ΔSNR Average ± SD** | **% Reduction ΔSNR from Mn^2+^ alone** | **TTEST P-Value (versus Mn^2+^ alone)** |
| CA1/2 | 0.46 ± 0.07 | 0.49 ± 0.07 | 53% | 0.0004 |
| CA3 | 0.38 ± 0.20 | 0.50 ± 0.20 | 59% | 0.0012 |
| DG | 0.33 ± 0.12 | 0.36 ± 0.12 | 56% | 0.0027 |
| SC | 0.29 ± 0.10 | 0.28 ± 0.10 | 55% | 0.0097 |
| Sub/EC | 0.26 ± 0.11 | 0.29 ± 0.11 | 70% | 0.0005 |
