# Supplementary material for: A hierarchy of manganese competition and entry in organotypic hippocampal slice cultures
Source: NMR Biomed. 2021 Feb 3;34(4):e4476. doi: 10.1002/nbm.4476 (PMC7988546; doi:10.1002/nbm.4476)
Supplement: Supplementary file 2 — Supporting info item. [file NBM-34-e4476-s002.pdf]

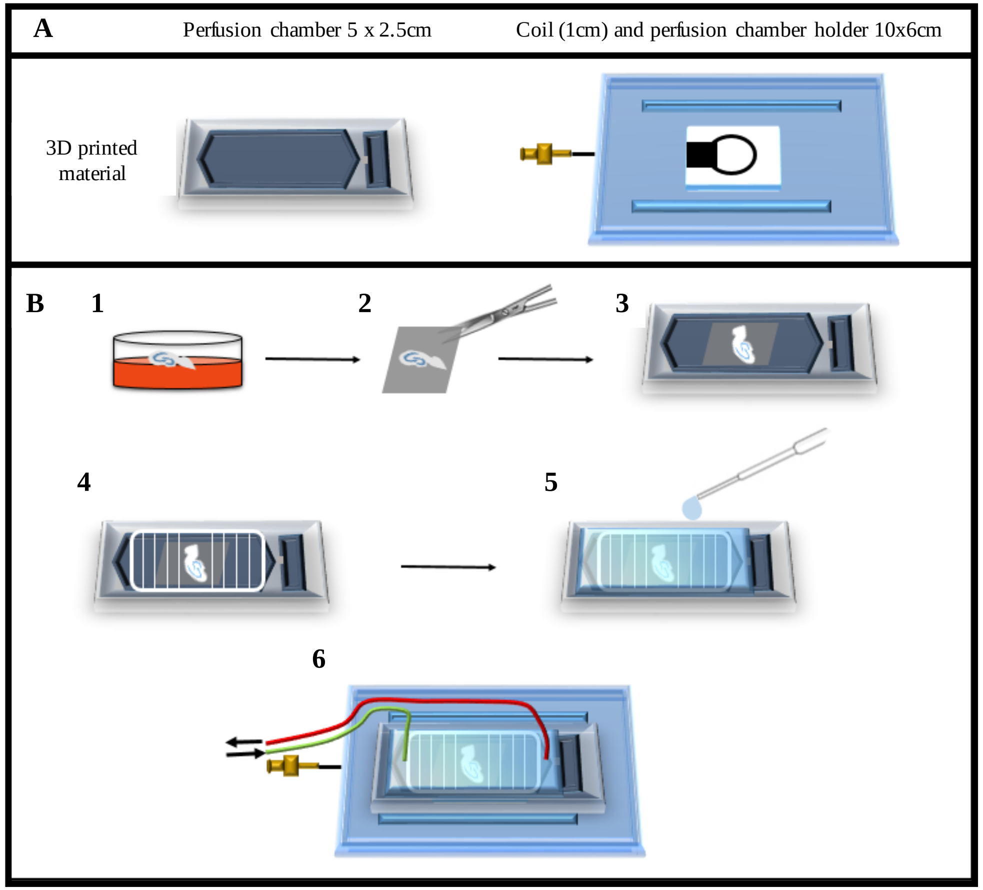
A
Perfusion chamber 5 x 2.5cm
Coil (1cm) and perfusion chamber holder 10x6cm
3D printed material
B
1
2
3
4
5
6

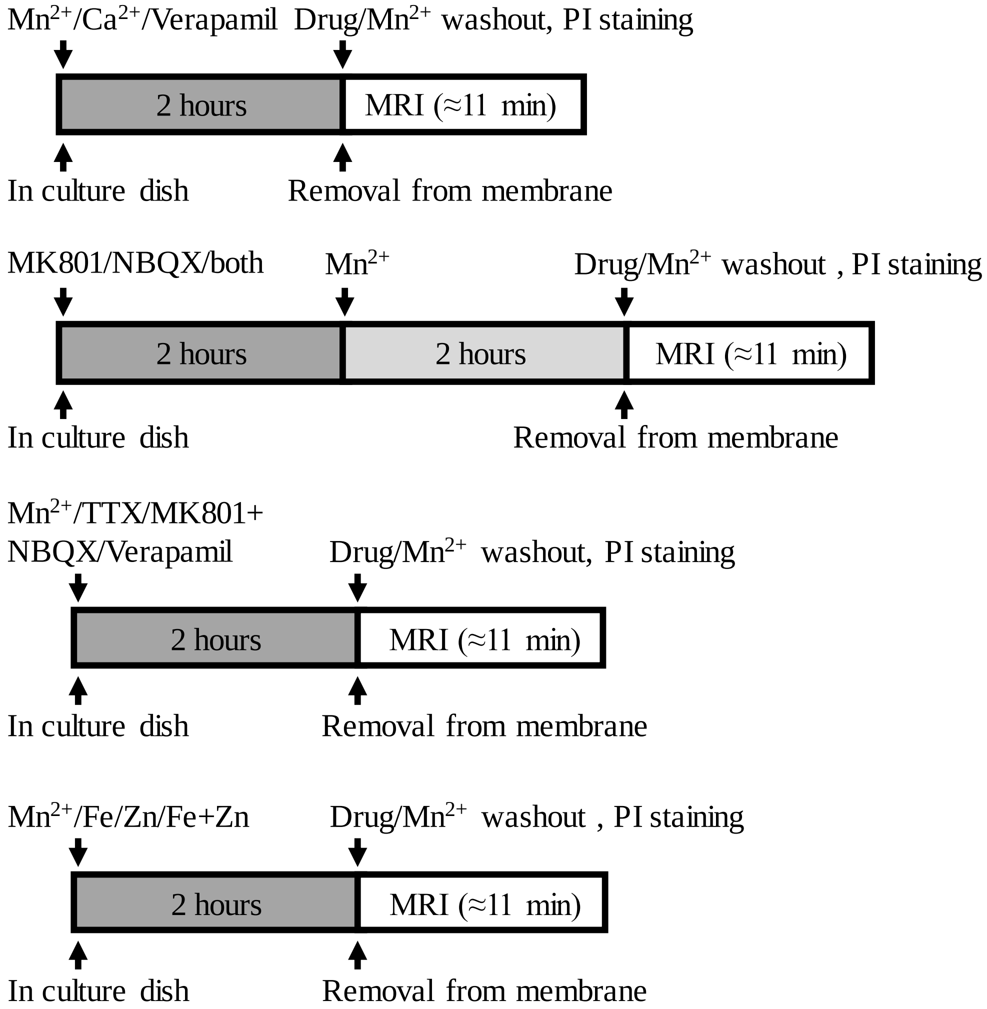
Mn2+/Ca2+/Verapamil
Drug/Mn2+ washout, PI staining
2 hours
MRI (≈11 min)
In culture dish
Removal from membrane
MK801/NBQX/both
Mn2+
Drug/Mn2+ washout , PI staining
2 hours
2 hours
MRI (≈11 min)
In culture dish
Removal from membrane
Mn2+/TTX/MK801+
NBQX/Verapamil
Drug/Mn2+ washout, PI staining
2 hours
MRI (≈11 min)
In culture dish
Removal from membrane
Mn2+/Fe/Zn/Fe+Zn
Drug/Mn2+ washout , PI staining
2 hours
MRI (≈11 min)
In culture dish
Removal from membrane

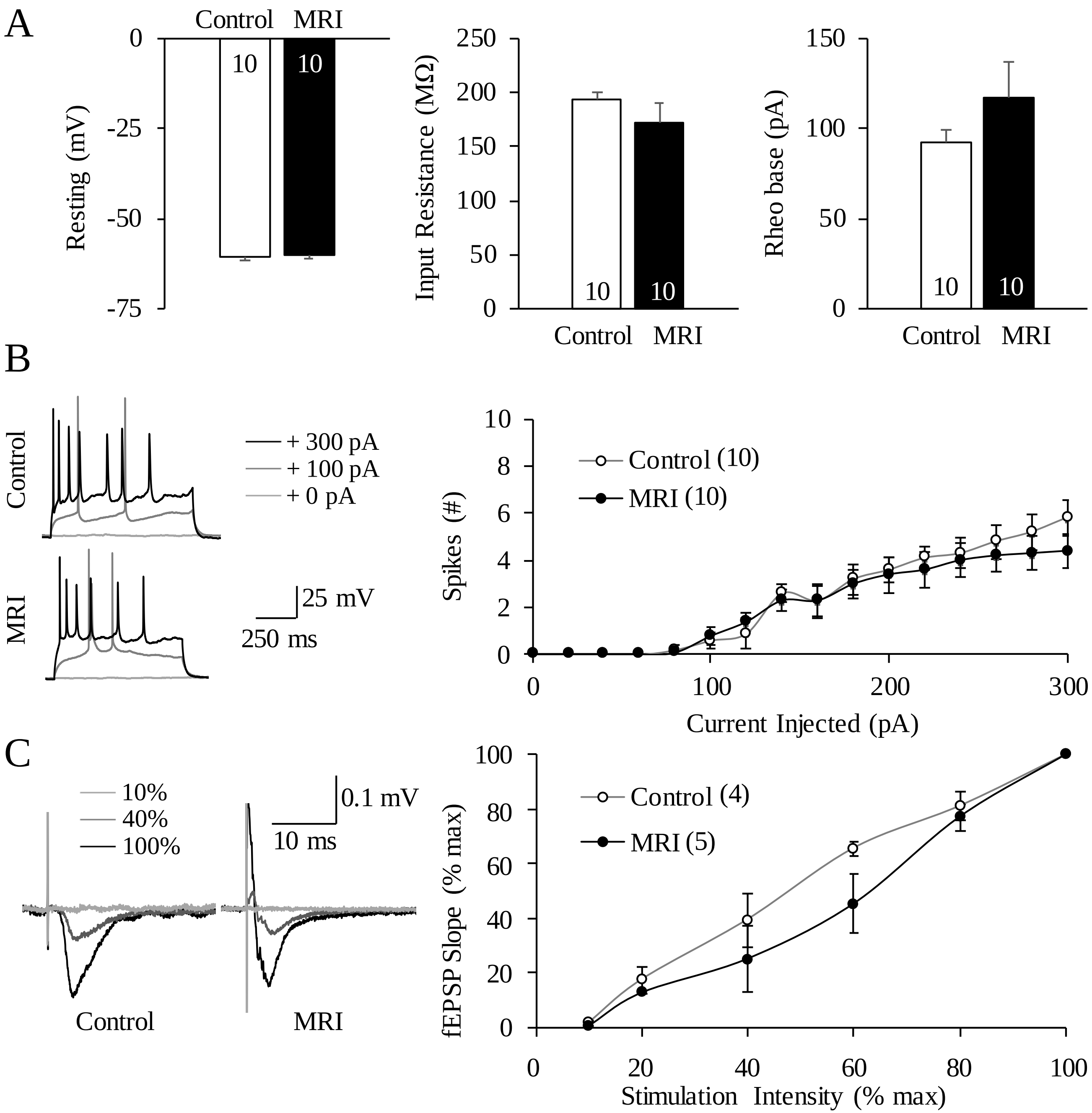
A
Control
MRI
Resting (mV)
0
-25
-50
-75
10
10
Input Resistance (MΩ)
250
200
150
100
50
0
Rheo base (pA)
150
100
50
B
Control
MRI
+ 300 pA
+ 100 pA
+ 0 pA
25 mV
250 ms
Spikes (#)
Control (10)
MRI (10)
Current Injected (pA)
0
100
200
300
C
10%
40%
100%
0.1 mV
10 ms
fEPSP Slope (% max)
Control (4)
MRI (5)
Stimulation Intensity (% max)
0
20
40
60
80
100

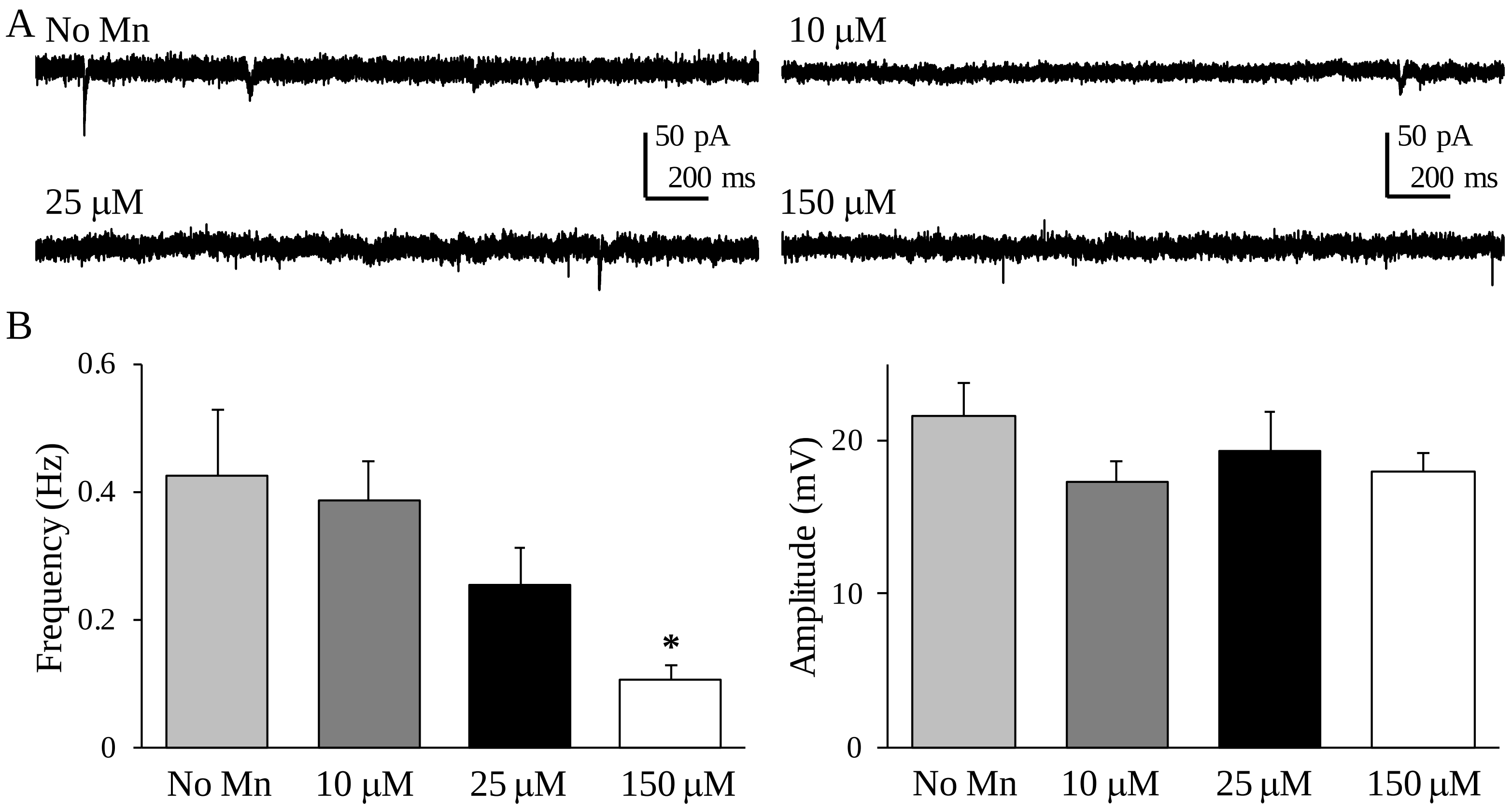

A
No Mn
10 μM
50 pA
200 ms
25 μM
150 μM
B
Frequency (Hz)
0.6
0.4
0.2
0
*
No Mn
10 μM
25 μM
150 μM
Amplitude (mV)
20
10
0

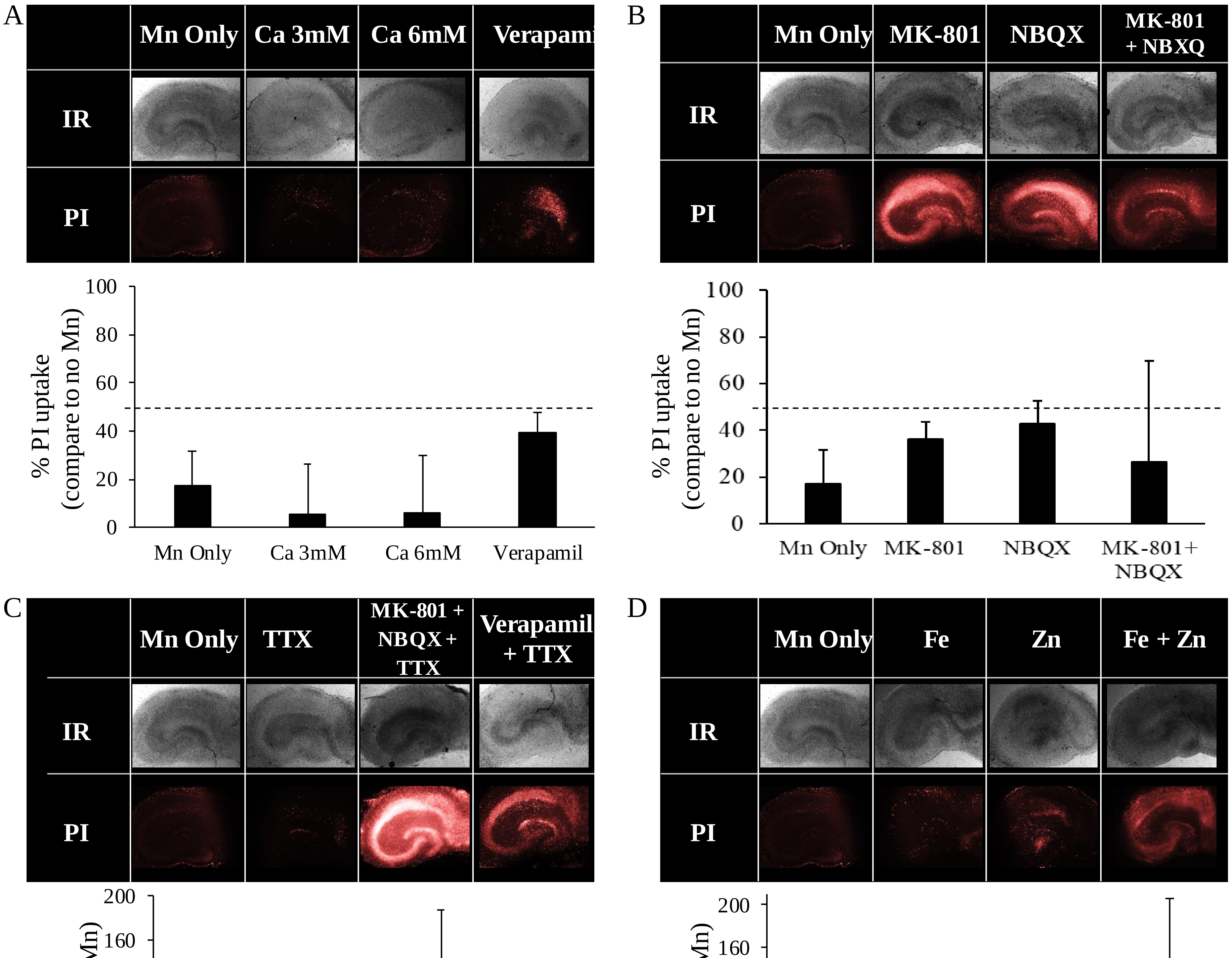
A
Mn Only
Ca 3mM
Ca 6mM
Verapami
IR
PI
% PI uptake (compare to no Mn)
100
80
60
40
20
0
Mn Only
Ca 3mM
Ca 6mM
Verapamil
B
Mn Only
MK-801
NBQX
MK-801 + NBXQ
IR
PI
% PI uptake (compare to no Mn)
100
80
60
40
20
0
Mn Only
MK-801
NBQX
MK-801+ NBQX
C
Mn Only
TTX
MK-801 + NBQX + TTX
Verapamil + TTX
IR
PI
200
160
D
Mn Only
Fe
Zn
Fe + Zn
IR
PI
200
160

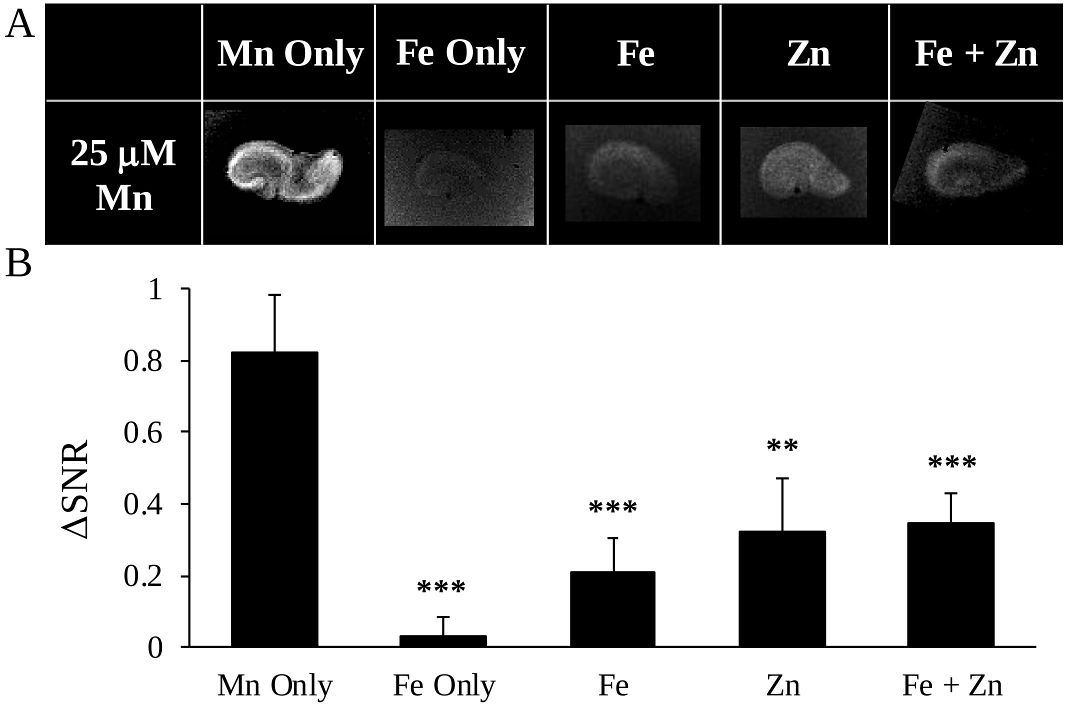
A
Mn Only
Fe Only
Fe
Zn
Fe + Zn
25 μM Mn
B
1
0.8
0.6
0.4
0.2
0
ΔSNR
***
***
**
***
Mn Only
Fe Only
Fe
Zn
Fe + Zn
